# Gaps in diagnosis and unmet healthcare needs in male sexual dysfunction and chronic health conditions: insights from a German population-based study

**DOI:** 10.1186/s12610-025-00293-y

**Published:** 2025-11-20

**Authors:** Elena Mühle, Selina M. Kronthaler, Carlotta Oesterling, Tatjana Tissen-Diabaté, Klaus M. Beier, Jörg Neymeyer, Thorsten Schlomm, Laura Hatzler

**Affiliations:** 1 Charité – Universitätsmedizin Berlin, Institute of Sexology and Sexual Medicine, Berlin, Germany; 2 Charité – Universitätsmedizin Berlin, Institute of Social Medicine, Epidemiology and Health Economics, Berlin, Germany; 3 Charité – Universitätsmedizin Berlin, Department of Urology, Berlin, Germany

**Keywords:** Male sexual dysfunction, Chronic health conditions, Mental health conditions, Help-seeking, Preferences, Therapy, Erectile dysfunction, Sexual health, Health care use, Diagnosis prevalence, Dysfonction sexuelle masculine, Pathologies chroniques, Troubles mentaux, Recours aux soins, Préférences thérapeutiques, Prise en charge, Dysfonction érectile, Santé sexuelle, Accès aux soins de santé, Prévalence du diagnostic

## Abstract

**Background:**

Chronic health conditions (CHC), both somatic and mental, increase the risk of sexual dysfunctions (SD) in men, which are associated with reduced quality of life. Despite existing guidelines, help-seeking remains low due to barriers such as shame and limited access, with many turning to anonymous sources. Representative data on care pathways and treatment preferences across SD domains and CHC subgroups is lacking. This study examines SD diagnoses, help-seeking, and treatment preferences in men with and without CHC meeting ICD-11 SD criteria to inform more tailored care.

**Results:**

Of all *N* = 1815 (unweighted *N* = 1787) cis-men, *n* = 265 (16.6%) fulfilled positive ICD-11 SD criteria and were included in this study (mean age 49.2; SD = 16.9 years). CHC were present in 74.1% of men, of whom 23.2% had self-reported SD. While the internet remains the most used information source, urologists were the preferred information sources and dialogue partners for sexual health concerns. Men with mental health conditions (MH +) valued psychotherapists and psychiatrists more highly in this regard. Shame was the most cited barrier to help-seeking, particularly present in men with MH + , with 50.4%. Only 46.6% of men with SD symptoms meeting the ICD-11 criteria reported an SD diagnosis. Previous therapy was rare, with 3-4 months of waiting times. Medication was the most commonly used treatment in the past. As preferred treatment, men with CHC prioritized medication (42.6% vs. 36.7% in men without CHC), whereas men without CHC also favored relaxation methods (29.3%). Desired treatment goals included improved sexual and relationship satisfaction. Digital tools such as apps or websites were also of interest, with reimbursement considered essential.

**Conclusion:**

Despite the high burden, SD diagnoses remain rare, and help-seeking behaviors vary, especially between men with somatic versus mental CHC. Regular healthcare contact may offer opportunities to address sexual health in trusted settings. Interventions should target both sexual and relationship satisfaction. Digital solutions can help close treatment gaps and improve access to specialized care. However, given low interest and adherence—particularly among men with CHC—tailored approaches are essential. Reimbursement within the German healthcare system is needed to lower financial barriers.

**Supplementary Information:**

The online version contains supplementary material available at 10.1186/s12610-025-00293-y.

## Résumé

**Contexte:**

Les affections chroniques (CHC) augmentent le risque de dysfonctions sexuelles (SD) chez les hommes et affectent leur qualité de vie. Malgré des lignes directrices existantes, la recherche d’aide reste faible.

**Résultats:**

Parmi *N* = 1815 hommes, *n* = 265 (16.6%) répondaient aux critères de SD selon la CIM-11 (âge moyen: 49.2 ans, SD 16.9). Des CHC étaient présentes chez 74.1%, dont 23.2% déclaraient une SD. Internet était fréquemment utilisé, mais les urologues restaient les interlocuteurs préférés. La honte était le principal obstacle à la recherche d’aide (50.4% chez les MH+). Seuls 46,6% avaient reçu un diagnostic de SD. Les traitements antérieurs étaient rares, avec des délais d’attente de 3-4 mois. Les médicaments étaient les plus utilisés et préférés, notamment par les hommes avec CHC (42.6% contre 36.7% sans CHC). Les hommes sans CHC privilégiaient aussi des méthodes de relaxation (29.3%). Les objectifs souhaités comprenaient une amélioration de la satisfaction sexuelle et relationnelle. Les outils numériques suscitaient de l’intérêt; leur remboursement était jugé essentiel.

**Conclusion:**

Malgré la charge élevée, les diagnostics de SD sont rares. La recherche d’aide varie selon le type de CHC. Les solutions numériques peuvent améliorer l’accès, mais requièrent des approches personnalisées et un remboursement systémique.

## Introduction

Sexual health encompasses physical, emotional, mental, and social well-being related to sexuality and goes beyond the mere absence of disease [1]. Sexual dysfunction (SD) manifests as a sexual problem in conjunction with severe sexual distress [2] and was found to correlate with reduced quality of life [3, 4] and psychological wellbeing [5]. Conversely, impairments of sexual health may contribute to mental health conditions such as depression and anxiety, reflecting a bidirectional relationship [6]. In Germany, 13.3% of men experienced SD within the past 12 months [7]. While a wide array of factors of biological and psychological nature have been associated with SD, for men in particular, somatic chronic health conditions (CHC) have been shown to pose a relevant risk factor for SD [8–13]. SD affects 63.6% of men with cardiovascular disease [8] and is up to three times more prevalent in men with diabetes, hypertension, or peripheral arterial disease [12]. The association between SD and CHC goes is postulated to be bidirectional. For instance, erectile dysfunction (ED) is recognized as an early marker of cardiovascular disease [14–16]. Research further indicates that mental CHC, such as depression and anxiety, represent a risk factor for SD [14, 17–19]. The effects from CHC on sexuality go beyond the direct consequences of the condition itself; they also include side effects of treatment as well as the impact of chronic disease on individuals' social lives, self-image, and body image [20]. In some cases, the CHC and its treatments were observed to enhance sexual function, e.g. dopaminergic therapy in Parkinson’s disease may increase libido or sexual behaviors [21].

With 60% of the German population affected by somatic or mental conditions [22], SD is a highly relevant issue and yet often overlooked in clinical care. Clinical guidelines emphasize individualized, multimodal care for SD. For example, the European Associations of Urology (EAU) and the American Society of Clinical Oncology (ASCO) recommend tailoring interventions – ranging from lifestyle changes to medical or psychological interventions – to each patient’s needs, comorbidities, and goals [23, 24]. In this context, lifestyle modifications (e.g., physical activity, weight management, smoking cessation) constitute an important component of care by targeting underlying risk factors that may contribute to the development or progression of SD [25]. Given the availability of clear clinical guidelines, SD appears to be an addressable problem within the healthcare system. Given the biopsychosocial etiology of sexual dysfunctions [26], current clinical guidelines recommend multimodal treatment approaches delivered by multidisciplinary teams in sexology or sexual medicine, with particular emphasis on psychosocial interventions such as cognitive-behavioral therapy [23, 24]. Robust evidence supports the efficacy of sex therapy, and couple-based approaches delivered in both digital and in-person formats [27–29]. Nonetheless, SD frequently remains undiagnosed [30, 31] and consequently untreated. Furthermore, in contrast to the biopsychosocial understanding of SD, pharmacotherapy remains the most frequently utilized option: The proportion of men taking oral medication for their SD differs substantially between studies, with findings ranging from 3 to 40% [2, 11, 12, 32–34].

A possible reason for the apparent gap in diagnosis and treatment may be the overall low help-seeking behavior among affected men. Previous studies analyzing such behavior have reported that only 9–43% of men with SD seek professional care [11, 32, 33, 35, 36]. Relevant influencing factors, among others, may involve the feeling of discomfort or embarrassment towards talking about sexuality [32, 33, 36–38], availability and accessibility [32, 36], and affordability [32, 36, 37] of consultation or certain treatments.

In recent years, a growing number of digital interventions have been developed to treat SD. These treatment programs are grounded in cognitive-behavioral and sex therapy frameworks while including components such as psychoeducation, meditative practices, partnered focus exercises, emotional counseling and guided pelvic floor training [29, 39, 40]. These interventions hold the potential to reduce the existing treatment gap by addressing both structural and individual barriers through the provision of low-threshold, accessible treatment options. However, evidence suggests that adherence among patients with CHC remains limited [41], which may substantially compromise the effectiveness of otherwise well-designed digital interventions. This highlights the importance of studying patient preferences to tailor future interventions to personal needs of individual patient groups.

While several studies have examined help-seeking behavior among men with SD, studies investigating their preferred pathways within the healthcare system, their preferred treatment approaches, and specific expectations regarding treatment goals remain scarce. Gaining insights into these preferences may help facilitate access to therapy and ensure that appropriate support is made more readily available to a larger number of men. Furthermore, no studies to date have systematically compared different male patient subgroups, such as patients with or without CHC or mental health conditions, in terms of their help-seeking behaviors and healthcare preferences. This information about specific healthcare needs in disease-specific subgroups is needed to tailor access to help and therapeutical options to respective subgroups. The present study aims to assess the rate of self-reported clinical SD diagnoses and to investigate help-seeking behaviors and healthcare preferences among men meeting ICD-11 criteria for SD, with particular consideration of comorbid CHC and mental health status.

## Patients and methods

### Study design and participants

**Fig. 1:**
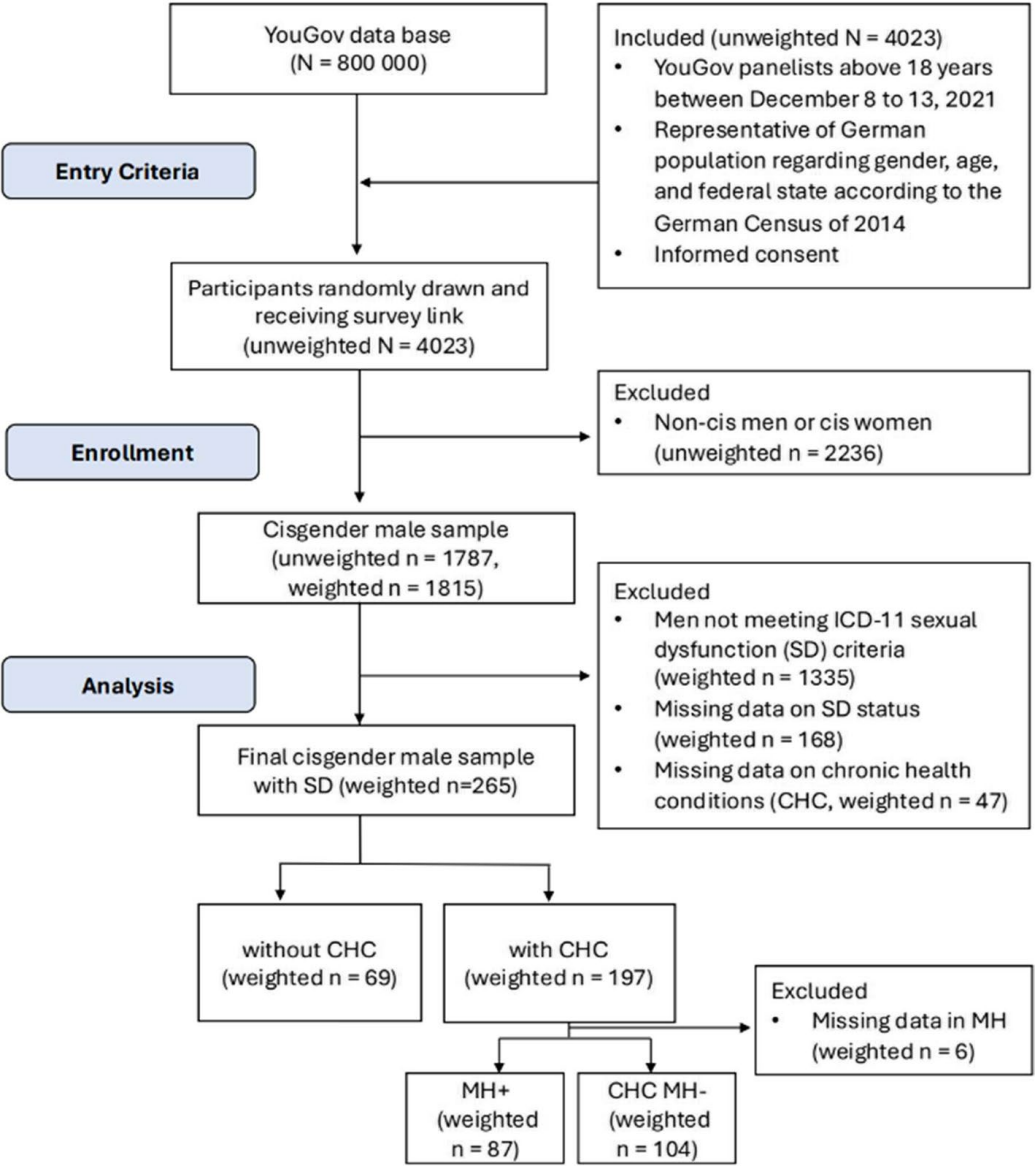
Flowchart of study participants (weighted numbers).This flowchart illustrates patient enrollment, including inclusion and exclusion criteria, and the final analytic sample. Abbreviation: SD, sexual dysfunction. CHC, chronic health conditions. MH +, comorbid mental CHC. CHC MH-, CHC excluding MH

In this cross-sectional study, data from a population-based survey conducted in 2021 via YouGov Deutschland is analyzed. Participants representative of the German population in terms of age, gender, and federal state were recruited as part of a research project funded by the Patient and Stakeholder Engagement (PSE) grant. The YouGov Germany panel consists of 800.000 individuals. Participants were invited via email with a survey link until a total of *N* = 4000 respondents was reached. Panelists were randomly invited to active studies based on the predefined quotas to ensure representativeness. To further enhance representativeness, regarding age, gender, and federal state, survey weights based on the 2014 Microcensus were applied by YouGov to the data [42]. The present study excluded women and transgender and gender diverse individuals. The sample of cis-women (*n* = 1985) was examined in the work by Kronthaler et al. [43]. Preliminary analyses have already been conducted on the sample of transgender and gender-diverse individuals (*n* = 223) [44], with further analyses planned for this population. This study reports data exclusively from cisgender men currently meeting ICD-11 criteria for SD. See Fig. 1 for an overview of participant flow. The comparison of data with the Microcensus 2014 and additional details on the recruitment process and data quality assurance are described in the work of Kronthaler et al., focusing on healthcare needs in women [43]. This study adhered to the Strengthening the Reporting of Observational Studies in Epidemiology (STROBE) guidelines for cross-sectional studies, see Additional Table 1 for more information.

**Table 1:** Characteristics of the study population with SD by CHC and MH status, and CHC subgroups

	**no CHC**	**CHC**	**MH + **	**CHC MH-**	**CV + **	**UR + **	**IN + **	**CA + **	**PA + **	**NE + **
**N**	69	197	87	104	123	54	42	24	36	15
**Sociodemographics, n (%)**										
Age, mean (SD), y	42.9 (14.8)	51.5 (17.1)	49.5 (15.9)	52.5 (17.8)	56.5 (15.1)	47.6 (20.3)	53.7 (17.5)	54.4 (20.7)	51.0 (17.8)	45.3 (17.2)
**Age groups, y**										
18–30	14 (20.3)	34 (17.3)	16 (18.0)	17 (16.7)	12 (9.5)	17 (31.2)	7 (19.9)	3 (13.9)	8 (18.8)	3 (21.8)
31–40	18 (25.7)	25 (12.6)	10 (11.0)	15 (14.6)	8 (6.6)	8 (15.6)	6 (15.8)	6 (26.0)	2 (5.1)	3 (21.3)
41–50	16 (23.9)	28 (14.2)	17 (19.8)	11 (10.3)	17 (13.7)	5 (9.8)	3 (8.3)	0 (0)	5 (12.4)	2 (13.7)
51–65	16 (22.7)	57 (29.2)	30 (34.2)	28 (26.5)	42 (34.4)	6 (10.6)	11 (30.7)	2 (9.8)	13 (31.1)	6 (37.2)
> 65	5 (7.5)	53 (26.8)	15 (16.9)	33 (32.0)	44 (35.8)	18 (33.0)	9 (25.3)	12 (50.4)	14 (32.6)	1 (5.9)
Education, ≥ 12 years	39 (56.2)	87 (44.2)	37 (42.7)	48 (46.1)	50 (40.8)	24 (44.5)	18 (49.2)	11 (43.6)	16 (38.7)	7 (47.6)
**Monthly net income, EUR**										
< €2500	44 (66.4)	121 (66.4)	62 (76.6)	57 (58.0)	75 (65.7)	30 (64.1)	16 (53.4)	12 (58.1)	26 (66.4)	6 (38.8)
€2500–5000	19 (29.0)	52 (28.5)	19 (23.4)	32 (32.5)	32 (27.9)	13 (26.9)	13 (43.6)	7 (36.5)	10 (25.3)	8 (54.1)
> €5000	3 (4.6)	9 (5.1)	0 (0)	9 (9.5)	7 (6.4)	4 (9.0)	1 (3.0)	1 (5.5)	3 (8.3)	1 (7.1)
**Employed**	47 (69.9)	84 (43.6)	27 (31.8)	56 (54.9)	44 (36.5)	25 (47.6)	17 (46.5)	10 (42.4)	18 (42.6)	6 (42.8)
**Religious**	43 (64.4)	119 (62.5)	50 (58.6)	66 (66.5)	74 (61.0)	34 (67.3)	21 (58.6)	16 (69.1)	24 (59.2)	11 (71.9)
**Currently in a relationship**	46 (66.2)	112 (57.1)	44 (50.4)	66 (63.0)	74 (59.9)	27 (49.5)	21 (59.6)	18 (74.5)	26 (61.6)	8 (50.5)
**Heterosexual**	63 (95.2)	144 (77.4)	63 (77.5)	76 (77.2)	97 (80.6)	36 (68.0)	21 (63.1)	19 (86.9)	31 (75.9)	9 (65.0)
**With migration background**	15 (22.1)	22 (11.2)	12 (14.3)	10 (9.3)	10 (8.4)	7 (13.4)	4 (10.5)	3 (12.6)	4 (10.4)	2 (14.3)
**Children, ≥ 1 in same household**	20 (29.7)	46 (23.4)	23 (26.2)	22 (21.4)	27 (21.9)	14 (25.2)	10 (26.9)	5 (21.8)	10 (23.4)	5 (29.2)
**Household size, ≥ 2**	51 (73.9)	150 (76.3)	63 (72.5)	83 (80.0)	91 (73.8)	45 (83.8)	25 (69.1)	21 (85.6)	33 (79.4)	12 (79.3)
**Doing the majority of housework**	17 (26.1)	53 (27.5)	28 (32.7)	23 (22.5)	36 (30.4)	14 (26.4)	13 (38.5)	3 (12.5)	15 (35.1)	5 (33.5)
**Primary caregiver**	4 (5.4)	15 (7.7)	6 (6.5)	9 (9.0)	12 (9.8)	6 (12.0)	3 (9.2)	1 (4.0)	1 (2.5)	2 (14.7)
**Living in urban area**	24 (34.2)	83 (42.0)	33 (37.6)	48 (46.2)	55 (44.8)	20 (36.3)	14 (38.9)	13 (53.7)	21 (50.8)	9 (61.1)
**Behavioral risk factor, n (%)**										
Medication for chronic condition	8 (11.5)	109 (55.7)	54 (63.1)	52 (49.5)	81 (66.4)	28 (51.6)	23 (55.5)	13 (54.1)	22 (63.3)	9 (58.0)
Alcohol consumption^c^	22 (32.8)	52 (27.0)	20 (23.1)	31 (29.5)	35 (28.4)	10 (18.2)	10 (29.0)	3 (11.7)	13 (30.5)	4 (26.3)
Smoking	32 (47.9)	104 (53.3)	46 (53.6)	58 (55.5)	67 (55.2)	31 (56.6)	11 (32.4)	13 (53.9)	18 (42.7)	13 (81.6)
Low physical activity^c^	24 (34.7)	57 (29.0)	25 (28.3)	30 (28.4)	30 (24.7)	12 (22.4)	13 (34.8)	7 (27.8)	11 (25.5)	3 (20.5)
Sexual discrimination	1 (1.4)	5 (2.7)	2 (2.3)	3 (3.2)	4 (3.5)	4 (8.0)	0 (0)	1 (4.6)	2 (4.9)	1 (7.5)
**Sexual Behavior, n (%)**										
Masturbation^b^	43 (64.5)	118 (60.8)	57 (66.7)	60 (57.7)	77 (63.2)	25 (45.8)	23 (66.7)	12 (49.2)	28 (66.0)	7 (46.1)
Partnered sexual activity^b^	26 (38.6)	43 (21.9)	17 (19.6)	24 (22.9)	22 (18.4)	13 (23.3)	11 (32.6)	4 (16.8)	6 (14.3)	3 (19.7)
Sexual trauma^b^	1 (1.4)	13 (6.5)	7 (7.8)	6 (5.7)	9 (7.1)	7 (12.0)	3 (9.1)	2 (7.7)	4 (10.0)	3 (22.3)
Spending time in close relationships^b^	20 (29.9)	67 (35.2)	23 (27.0)	44 (42.8)	39 (32.3)	20 (37.3)	12 (35.5)	10 (41.4)	17 (39.6)	3 (18.1)

This table presents the characteristics of participants with sexual dysfunction (SD) stratified by overall chronic health condition (CHC) and mental health-related CHC status, and comorbid CHC subgroups. Categorical variables are presented as n (%); age is presented as mean (SD). Weighted frequencies and means are shown

*Abbreviation: SD* Sexual dysfunction, *CHC* Chronic health conditions. MH +, comorbid mental CHC. CHC MH-, CHC excluding MH. CV +, comorbid cardiovascular and metabolic CHC. UR +, comorbid urological CHC. IN +, comorbid infectious and inflammatory CHC. CA +, comorbid cancer CHC. PA +, comorbid pain-related CHC. NE +, comorbid neurological CHC. OR, odds ratio. NA, not applicable

^a^Weighted frequencies

^b^In the past 12 months

^c^ < once per week

### Questionnaire development with patient and public involvement

To ensure patient relevance of the study design, representatives from non-profit organizations representing vulnerable populations at increased risk of experiencing SD were involved through a patient and public involvement process. Their input informed the selection of validated questionnaires and the development of new patient-relevant items. From July to December 2021, a total of five advisory board members and three co-researchers were involved in developing the survey questionnaire. Further details on this process are described in the work of Kronthaler et al. [43].

The final questionnaire incorporated validated instruments: the Female Sexual Distress Scale–Desire/Arousal/Orgasm by Derogatis et al. [45], the Screening for Sexual Problems in Men (SSP-M) by Velten and Zarski [46], and the Relationships Questionnaire–2 (RQ-2) [47]. Beyond these validated scales, additional items were included to gather information on sociodemographic factors, sexual health status, self-reported diagnoses, biopsychosocial protective and risk factors, patterns of help-seeking behavior, and healthcare needs. Within the sexual health section, a specific question regarding the participant’s awareness of current or past sexual difficulties was used as a screening filter to direct respondents to subsequent help-seeking-related questions. Additional sociodemographic and health-related items were already available through YouGov and purchased for use in this study. A more detailed description of the questionnaire development and full codebook is provided in Kronthaler et al. [43].

### Assessment of self-reported received diagnosis

Participants’ self-reported clinical diagnoses of SD were obtained, complementing the self-reported disease items obtained from YouGov. Participants were asked to indicate any formal diagnoses they had received from physicians or psychologists within the healthcare system. The list included both somatic conditions — later used to classify individuals with CHC, as detailed below — and SD diagnoses such as hypoactive sexual desire disorder (HSDD), ED, orgasmic disorder (OD), and premature ejaculation (PE). The response format was multiple choice, allowing participants to select all diagnoses that applied to them. Binary variables were calculated for different SD diagnosis prevalence subdomains and total SD diagnoses.

### Assessment of help-seeking behavior

Help-seeking behavior for sexual problems was assessed using six measures: received treatment (yes/no), time to access, accessed information source (e.g., internet, urologist), accessed dialogue partners (e.g., partner, psychotherapist), received offerings (e.g., medication, relaxation techniques), and barriers (e.g., shame, availability). All measures, except for barriers, were obtained only from participants who self-reported current or past sexual problems. Time to access treatment was rated on a 5-point Likert-scale (1 = “under one month”, 2 = “1–2 months”, 3 = “3–4 months”, 4 = “5–6 months”, 5 = “longer than 6 months”). The remaining items consisted of multiple-choice questions that enabled the selection of multiple responses. Full details on all items can be found in the work of Kronthaler et al. [43].

### Assessment of healthcare needs

Data on healthcare needs was obtained through a set of eight items presented to all participants. Each item was introduced by the sentence: “Assume you have sexual problems and feel distressed by them”. The items included: preferred information source (e.g., internet, urologist), preferred dialogue partners (e.g., partner, psychotherapist), treatment goals (e.g., sexual satisfaction, more orgasms), preferred offerings (e.g., medication, relaxation techniques), favored future developments (e.g., new drugs, new surgery), preferred design of digital offers (e.g., possibility to contact experts, reimbursement), preferred expert contact (appointments for exchange via chat, feedback via email) and willingness to pay for treatments. Preferred design of digital offers and expert contact were rated on a 10-point Likert scale ranging from 1 (“not at all important”) to 10 (“extremely important”). Willingness to pay for an effective solution was assessed using a categorical variable with nine response options: 0 = “nothing,” 1 = “1–50 €,” 2 = “51–100 €,” 3 = “101–150 €,” 4 = “151–200 €,” 5 = “201–300 €,” 6 = “301–400 €,” 7 = “401–500 €,” and 8 = “more than 500 €.” For reporting purposes, these were subsequently grouped into five categories: *Nothing*, < *€50*, *€51–100*, *€101–300*, and > *€300*, and descriptively summarized using frequencies (n, %). For the items treatment goals and favored future developments, participants were asked to select the three most important options. All other items were designed as multiple-choice questions, allowing participants to select all applicable response options.

### Chronic health conditions

Participants were categorized into two groups: those with CHC (CHC) and those without (no CHC). To further differentiate among individual CHC, subgroups were formed, irrespective of comorbidity with other conditions: mental health (MH +; i.e., depression, anxiety, autism, post-traumatic stress disorder, premenstrual dysphoric disorder, other mental health conditions), cardiovascular and metabolic (CV +; i.e., arteriosclerosis, cardiovascular disease, hypertension, diabetes, dyslipidemia, osteoporosis), urologic conditions (UR +; i.e., incontinence, pelvic floor dysfunctions, urinary tract infections, lichen sclerosis, infertility longer than 6 months); infections/inflammations (IN +; i.e., rheumatoid arthritis, joint inflammation, rheumatism, psoriasis, sexually transmitted infections (STI’s), HIV or AIDS), cancer (CA +; i.e., prostate, breast, other), pain (PA +; i.e., chronic pain, chronic pelvic pain, chronic bladder pain syndrome) and neurological conditions (NE +; i.e., dementia, Alzheimer’s disease, Parkinson’s disease, stroke, epilepsy, cerebral palsy, multiple sclerosis). In addition, participants with CHC were further classified into those with exclusively somatic conditions but no mental health CHC (CHC MH −), and those with a mental health condition, irrespective of somatic CHC comorbidity (MH +).

### Statistical assessment

Statistical analyses were performed using R (version 4.3.2). All frequencies, including the total number of participants, were reported as weighted values. This exploratory analysis used weighted logistic regression to examine associations between CHC and binary outcomes related to clinical diagnosis of SD, help-seeking behaviour and healthcare needs. Odds ratios (ORs) are presented with 95% confidence intervals (CI). For ordinal variables with four or more categories, the median and interquartile range (IQR) were calculated. Results are presented for the subgroups CHC, no CHC, MH +, CHC MH-, UR +, IN +, CA +, PA + and NE +. Responses marked as 'Not specified' or 'Not answered' were treated as missing. No imputation of missing values was performed. All analyses were exploratory, with no significant thresholds applied and no correction for multiple testing.

## Results

### Sample characteristics

Of all *N* = 1815 (unweighted *n* = 1787) cisgender men, *n* = 265 (16.6%) met the ICD-11 criteria for SD and were included in the study. Additional information on completion rates is presented in the work by Kronthaler et al., currently in press [43]. The mean age of participants was 49.2 years (SD 16.9 years). CHC were present in 74.1% (*n* = 197). The most common CHC subgroup was CV + (*n* = 123, 46.4%), followed by MH + (*n* = 87, 32.8%), UR + (*n* = 54, 20.4%), and IN + (*n* = 42, 15.8%). The prevalence of all other subgroups was below 15%. When stratified by mental health status, *n* = 87 (45.5%) men were classified as MH +, and *n* = 104 (54.5%) men as CHC MH-. Further information on prevalences and comorbidities of CHC subgroups can be found in the unpublished work by Kronthaler et al. on male participants. In the past 12 months, 26.2% of men reported having engaged in partnered sexual activity, and 61.7% reported having masturbated. For further descriptive statistics, see Table 1.

### Prevalence of diagnosis of SD

Among men currently meeting ICD-11 criteria for SD, only about half (46.6%) had received a formal diagnosis in the past. Detection rates were highest among participants with comorbid mental health conditions (53.5%), indicating better recognition compared to men with SD but without CHC (OR 2.36, 95% CI 1.15–4.84). Among the different SD diagnoses domains, ED showed the highest detection rates. For detailed prevalence of SD diagnoses, see Table 2.

**Table 2:** Prevalence of any and individual clinical SD diagnoses in men with SD, by CHC status

	**All men with SD**	**No CHC**	**CHC**	**OR for the diagnosis in CHC vs. no CHC (95% CI)**	**MH + **	**OR for the diagnosis in MH + vs. no CHC (95% CI)**	**CHC MH-**	**OR for the diagnosis in CHC MH- vs. no CHC (95% CI)**
	*N* = 265	*N* = 69	*N* = 197		*N* = 87		*N* = 104	
**SD (overall)**	115 (46.6%)	19 (32.6%)	97 (50.8%)	2.13 (1.12–4.06)	46 (53.5%)	2.36 (1.15–4.84)	51 (48.7%)	1.96 (0.98–3.92)
**Hypoactive sexual desire dysfunction (HSDD)**	32 (13.0%)	2 (3.9%)	30 (15.6%)	4.55 (1.04–19.9)	15 (17.0%)	5.03 (1.09–23.3)	15 (14.5%)	4.16 (0.90–19.1)
**Erectile dysfunction (ED)**	83 (34.5%)	12 (22.0%)	72 (38.1%)	2.18 (1.04–4.58)	33 (39.3%)	2.3 (1.02–5.17)	38 (37.1%)	2.09 (0.95–4.60)
**Orgasmic dysfunction (OD)**	24 (9.7%)	5 (8.1%)	19 (10.2%)	1.28 (0.45–3.64)	7 (8.1%)	1 (0.29–3.37)	12 (12.0%)	1.53 (0.51–4.61)
**Premature ejaculation (PE)**	32 (13.2%)	7 (13.4%)	25 (13.2%)	0.99 (0.39–2.46)	15 (18.0%)	1.43 (0.53–3.83)	9 (9.2%)	0.66 (0.23–1.86)

This table shows the prevalence [n (%)] of any and individual clinical sexual dysfunction (SD) diagnoses among men with sexual dysfunction (SD), stratified by all men, chronic health conditions (CHC), and mental health-related CHC. Associations between CHC and SD diagnoses were assessed using weighted logistic regression (odds ratios [OR] with 95% confidence intervals [CI])

*Abbreviation: SD* Sexual dysfunction, *CHC* Chronic health conditions, *MH* + Comorbid mental CHC, *CHC MH-* CHC excluding MH, *OR* Odds ratio, *CI* Confidence interval

### Treatment access and barriers

Only a limited number of men with SD reported previous access to therapy for sexual problems (15.3%) with only slight differences between men without CHC (13.6%) and men with CHC (15.8%, OR 1.20, 95% CI 0.50–3.21, MH + 17.0%, CHC MH- 15.3%). Regarding the CHC subgroups, men with neurological conditions and urological conditions had highest rates of received treatment across subgroups (NE + 48.9%, UR + 30.2%). The median time to access treatment was 3 to 4 months in nearly all groups, except for men with IN +, for whom it was 1 to 2 months. Overall, the most frequently reported barrier to help-seeking was shame (no CHC 47.9%, CHC 34.8%), with 50.4% of men with mental health issues citing this barrier. In contrast, among men without mental health issues (28.3%) and urological conditions (30.6%), the effectiveness of treatment was the most frequently mentioned barrier. Other barriers varied considerably across subgroups. However, the following barriers were reported more frequently overall: Knowledge about effectiveness of treatments and -availability of treatments, lack of services sensitive to physical illnesses, fear of being discovered and fear of not being taken seriously. Further details on treatment access and the full list of reported barriers across CHC subgroups can be found in Table 3.

**Table 3:** Help-seeking behavior in men with SD among CHC status, MH status, and CHC subgroups

	**no CHC (*n* = 69)**	**CHC (*n* = 197)**	**OR (95% CI)**^**a**^	**MH + (*n* = 87)**	**CHC MH- (*n* = 104)**	**CV + (*n* = 123)**	**UR + (*n* = 54)**	**IN + (*n* = 36)**	**CA + (*n* = 24)**	**PA + (*n* = 42)**	**NE + (*n* = 15)**
**Treatment, n (%)**	50	162		77	83	103	41	26	20	38	13
Received Treatment	7 (13.6)	26 (15.8)	1.20 (0.50–3.21)	13 (17.0)	13 (15.3)	18 (17.9)	13 (30.2)	4 (14.3)	4 (19.6)	9 (24.1)	6 (48.9)
**Time to access n (%)**	7	26		13	13	18	13	4	4	9	6
Less than 1 month	2 (30.3)	2 (8.5)		2 (16.9)	0 (0)	1 (5.2)	0 (0)	1 (25.6)	0 (0)	0 (0)	0 (0)
1–2 months	1 (13.9)	9 (35.9)		4 (29.1)	5 (43.0)	6 (33.9)	5 (43.3)	2 (49.7)	1 (26.9)	3 (36.6)	4 (67.7)
3–4 months	1 (12.5)	6 (25.0)		1 (9.0)	5 (41.5)	5 (29.8)	4 (32.8)	1 (24.7)	2 (51.4)	1 (10.0)	0 (0)
5–6 months	2 (27.6)	2 (8.3)		2 (16.3)	0 (0)	1 (5.9)	1 (8.7)	0 (0)	0 (0)	2 (23.2)	1 (16.9)
Longer than 6 months	1 (15.8)	6 (22.2)		4 (28.7)	2 (15.6)	5 (25.2)	2 (15.1)	0 (0)	1 (21.7)	3 (30.1)	1 (15.3)
**Information sources, n (%)**	50	162		78	82	102	44	27	19	38	14
Internet	32 (64.5)	75 (45.9)	0.47 (0.24–0.89)	36 (46.0)	37 (45.1)	46 (45.2)	14 (31.8)	14 (52.1)	8 (40.3)	20 (51.6)	4 (29.6)
Literature	8 (16.2)	19 (11.6)	0.68 (0.28–1.73)	9 (11.7)	10 (11.9)	11 (10.5)	9 (21.5)	4 (14.4)	2 (10.0)	7 (19.1)	2 (15.7)
Partner(s)	14 (28.1)	38 (23.4)	0.78 (0.39–1.64)	22 (28.4)	15 (18.3)	22 (21.6)	11 (24.7)	7 (26.9)	5 (24.3)	6 (15.5)	4 (27.7)
Friend(s)	6 (12.3)	16 (9.8)	0.78 (0.30–2.26)	10 (13.0)	6 (7.2)	9 (8.9)	1 (2.5)	3 (10.4)	1 (5.5)	4 (11.0)	4 (29.1)
Support Groups	5 (10.2)	13 (8.0)	0.77 (0.27–2.48)	4 (5.2)	8 (9.7)	8 (7.9)	6 (14.8)	4 (14.9)	1 (5.1)	5 (13.6)	3 (24.3)
General practitioner	10 (19.9)	45 (27.7)	1.54 (0.73–3.51)	25 (32.1)	20 (24.4)	30 (29.3)	11 (25.1)	4 (13.9)	5 (24.0)	13 (33.0)	3 (22.3)
Urologist	16 (31.1)	76 (46.5)	1.93 (1.00–3.88)	34 (43.0)	41 (50.4)	51 (49.7)	26 (58.7)	8 (29.7)	13 (68.2)	22 (58.6)	8 (55.5)
**Dialogue partners, n (%)**	49	162		79	81	102	41	27	20	37	13
Partner(s)	23 (47.6)	66 (40.9)	0.76 (0.40–1.45)	37 (46.2)	29 (35.5)	45 (44.1)	11 (27.7)	11 (40.2)	9 (45.9)	15 (41.4)	3 (23.2)
Family	4 (8.0)	9 (5.6)	0.67 (0.21–2.62)	5 (6.2)	4 (5.0)	2 (2.0)	4 (9.5)	3 (11.4)	3 (14.3)	2 (5.5)	1 (8.0)
Friend(s)	8 (16.4)	19 (11.5)	0.66 (0.28–1.71)	13 (16.4)	6 (7.1)	10 (9.5)	4 (9.9)	5 (17.5)	2 (9.7)	5 (14.1)	2 (15.4)
Peer network	2 (4.3)	5 (3.1)	0.70 (0.15–4.76)	2 (2.5)	3 (3.7)	3 (2.8)	0 (0)	2 (7.1)	1 (4.7)	0 (0)	0 (0)
General practitioner	10 (20.2)	44 (27.1)	1.48 (0.70–3.37)	21 (26.9)	23 (28.0)	31 (30.6)	10 (23.9)	5 (18.1)	6 (28.2)	9 (23.3)	5 (38.9)
Urologist	12 (24.7)	67 (41.4)	2.15 (1.07–4.59)	29 (37.3)	37 (45.3)	45 (44.5)	24 (57.1)	8 (29.7)	12 (60.2)	19 (51.9)	7 (53.0)
Psychiatrist	2 (4.2)	17 (10.5)	2.68 (0.74–16.90)	16 (20.4)	1 (1.2)	8 (8.2)	3 (7.5)	2 (7.6)	1 (4.8)	3 (8.4)	2 (15.7)
Other physician	1 (1.9)	12 (7.1)	1.93 (1.00–3.88)	5 (5.8)	7 (8.6)	8 (8.2)	5 (13.0)	2 (8.1)	3 (15.0)	1 (2.5)	1 (9.4)
Psychotherapist	2 (4.1)	27 (16.6)	4.60 (1.31–29.01)	21 (26.0)	6 (7.8)	12 (11.8)	6 (14.9)	6 (20.5)	2 (12.3)	7 (18.9)	1 (8.2)
Police	0 (0)	3 (1.8)	NAb	1 (1.0)	2 (2.7)	1 (0.8)	2 (5.2)	2 (6.4)	2 (10.8)	0 (0)	0 (0)
Lawyer	1 (2.0)	1 (0.7)	0.33 (0.01–8.69)	0 (0)	1 (1.3)	1 (1.1)	0 (0)	0 (0)	1 (5.5)	0 (0)	0 (0)
**Received offerings, n (%)**	46	165		78	85	104	43	27	20	38	13
Urologist exam	5 (11.1)	43 (25.8)	2.78 (1.12–8.38)	20 (26.2)	21 (25.3)	32 (30.9)	17 (39.4)	6 (23.0)	9 (46.7)	12 (32.7)	4 (32.0)
Other physician exams	3 (6.1)	15 (9.0)	1.52 (0.46–7.16)	8 (10.2)	7 (8.2)	11 (10.5)	7 (16.5)	2 (6.6)	1 (4.6)	6 (15.6)	1 (8.2)
Systemic hormone therapy	1 (1.9)	8 (5.1)	0.13 (0.01—1.25)	3 (4.0)	5 (6.3)	3 (3.1)	4 (8.3)	1 (4.5)	2 (11.1)	1 (2.6)	1 (9.3)
Local hormone therapy	2 (4.7)	1 (0.6)	2.84 (0.46–75.77)	1 (1.3)	0 (0)	0 (0)	1 (2.4)	0 (0)	0 (0)	0 (0)	0 (0)
Medication	13 (28.4)	70 (42.5)	2.08 (1.05–4.35)	29 (37.0)	40 (47.9)	49 (47.0)	19 (43.9)	12 (44.0)	9 (46.4)	18 (47.2)	5 (40.2)
Surgery	0 (0)	5 (2.9)	NAb	4 (4.8)	1 (1.2)	3 (2.6)	3 (6.4)	0 (0)	3 (13.6)	2 (4.5)	0 (0)
Psychotherapy	3 (6.3)	11 (6.7)	1.07 (0.31–5.03)	10 (12.9)	1 (1.2)	5 (5.0)	1 (2.2)	2 (8.2)	1 (4.8)	1 (2.8)	1 (7.4)
Sex and couples therapy	3 (6.7)	6 (3.9)	0.56 (0.15–2.69)	3 (4.2)	3 (3.7)	4 (4.3)	4 (10.0)	1 (3.6)	1 (4.9)	2 (5.6)	1 (8.2)
Support groups	2 (4.2)	6 (3.6)	0.86 (0.19–6.29)	5 (6.5)	1 (1.1)	2 (1.9)	3 (7.3)	3 (10.9)	0 (0)	3 (8.0)	0 (0)
Relaxation techniques	3 (6.8)	5 (2.9)	0.41 (0.10–2.04)	4 (4.9)	1 (1.2)	3 (3.1)	2 (5.1)	0 (0)	1 (5.0)	1 (2.6)	0 (0)
Pelvic floor training aids	1 (2.0)	11 (6.6)	3.54 (0.62—83.89)	6 (7.2)	5 (6.2)	6 (5.5)	5 (12.3)	1 (2.8)	3 (14.7)	3 (9.2)	0 (0)
Physiotherapy	0 (0)	5 (2.8)	NA	2 (2.5)	3 (3.2)	5 (4.5)	3 (6.8)	0 (0)	0 (0)	3 (7.7)	1 (8.2)
Biofeedback	0 (0)	3 (1.8)	NA	0 (0)	3 (3.6)	3 (2.9)	1 (2.4)	0 (0)	1 (5.1)	0 (0)	0 (0)
Penis pump	3 (6.7)	20 (12.0)	1.90 (0.62–8.22)	14 (18.3)	5 (5.5)	12 (11.6)	8 (18.7)	5 (17.6)	2 (8.7)	4 (10.4)	1 (8.4)
TENS	1 (2.3)	2 (1.1)	NA	1 (1.2)	1 (1.1)	1 (0.9)	2 (4.4)	2 (6.9)	1 (4.6)	1 (2.6)	0 (0)
Body therapies (e.g., massage, osteopathy)	0 (0)	6 (3.4)	NA	3 (3.4)	3 (3.5)	2 (1.9)	2 (4.7)	1 (3.6)	2 (10.1)	1 (2.5)	0 (0)
Physical activity	11 (24.2)	24 (14.6)	0.54 (0.24–1.23)	15 (19.9)	8 (9.0)	13 (12.7)	8 (18.3)	4 (13.5)	3 (13.5)	4 (10.4)	2 (15.1)
Diet	3 (6.0)	24 (14.4)	2.62 (0.83 −12.26)	12 (15.7)	11 (12.5)	13 (12.4)	7 (15.6)	4 (13.3)	3 (13.7)	7 (18.5)	5 (38.8)
**Barriers, n (%)**	65	189		86	101	117	54	33	24	42	15
**Lack of knowledge regarding**											
contact persons	16 (24.0)	41 (21.4)	0.86 (0.45–1.72)	21 (24.1)	20 (19.8)	24 (20.4)	8 (14.6)	10 (31.1)	6 (22.7)	6 (15.1)	2 (12.0)
available treatments	8 (12.6)	40 (21.2)	1.86 (0.86–4.46)	18 (20.6)	22 (22.3)	26 (22.4)	11 (20.3)	10 (31.8)	7 (27.8)	8 (20.2)	4 (24.9)
effectiveness of treatments	10 (15.9)	50 (26.4)	1.89 (0.93–4.15)	20 (23.8)	29 (28.3)	36 (30.6)	15 (28.3)	7 (21.4)	8 (32.7)	13 (30.7)	5 (35.2)
**Lack of services…**											
in the region or too long waiting times	10 (15.4)	23 (12.1)	0.75 (0.34–1.75)	16 (19.1)	6 (6.4)	12 (10.6)	10 (17.7)	7 (20.4)	1 (5.1)	5 (11.8)	2 (15.0)
sensitive to mental health	11 (17.2)	27 (14.3)	0.81 (0.38–1.79)	19 (22.7)	8 (7.6)	17 (14.7)	8 (15.4)	9 (26.5)	1 (3.8)	8 (19.1)	2 (13.8)
sensitive to physical illnesses	7 (11.2)	39 (20.5)	2.05 (0.92–5.16)	22 (25.5)	17 (16.8)	27 (23.1)	14 (25.3)	10 (29.2)	6 (24.8)	13 (31.5)	6 (40.4)
sensitive to sexual orientation and gender identity	2 (2.8)	13 (6.9)	2.60 (0.66–19.25)	7 (8.5)	6 (5.7)	9 (8.0)	6 (11.6)	5 (13.9)	1 (3.8)	1 (2.6)	1 (7.0)
sensitive to culture and religion	7 (10.2)	9 (4.7)	0.43 (0.15–1.30)	5 (5.3)	4 (4.3)	5 (3.9)	4 (8.1)	1 (3.3)	3 (14.0)	1 (2.6)	2 (14.0)
**Intrapersonal**											
Lack of time	7 (11.3)	22 (11.7)	1.04 (0.45–2.70)	12 (14.2)	10 (10.0)	9 (8.0)	10 (17.7)	7 (22.4)	2 (8.5)	5 (12.8)	2 (12.0)
Shame	31 (47.9)	66 (34.8)	0.58 (0.33–1.03)	43 (50.4)	22 (21.4)	39 (33.3)	10 (19.3)	8 (25.0)	4 (18.1)	14 (32.8)	5 (33.8)
Avoidance due to difficulty of topics	18 (27.3)	38 (19.9)	0.66 (0.35–1.30)	22 (26.0)	15 (14.4)	25 (21.0)	9 (17.1)	7 (20.0)	3 (10.4)	9 (21.8)	4 (28.1)
Fear of being discovered	13 (19.9)	42 (22.4)	1.16 (0.59–2.40)	26 (29.9)	17 (16.6)	27 (22.9)	13 (23.8)	6 (19.7)	4 (14.7)	12 (27.9)	3 (21.0)
Fear of not being taken seriously	20 (30.0)	38 (20.0)	0.58 (0.31–1.12)	22 (25.5)	16 (15.9)	22 (19.0)	11 (20.9)	6 (17.8)	6 (25.1)	14 (34.3)	3 (21.1)
No “need to talk”	5 (8.3)	13 (7.1)	NAb	6 (6.4)	8 (7.9)	9 (7.7)	7 (12.8)	3 (8.0)	2 (7.0)	6 (14.2)	3 (20.5)

This table provides detailed data on help-seeking behavior among men with sexual dysfunction (SD), stratified by overall chronic health condition (CHC) and mental health-related CHC status, and comorbid CHC subgroups. Reported variables include received treatment, time to access, information sources, dialogue partners, received offerings, and barriers. Categorical variables are shown as weighted frequencies. Odds ratios (OR) with 95% confidence intervals (CI) are reported for the comparison of men with versus without CHC, calculated using weighted logistic regression

*Abbreviation*: *SD* Sexual dysfunction, *CHC* Chronic health conditions, *MH* + Comorbid mental CHC, *CHC MH-* CHC excluding MH, *CV* + Comorbid cardiovascular and metabolic CHC, *UR* + Comorbid urological CHC, *IN* + Comorbid infectious and inflammatory CHC, *CA* + Comorbid cancer CHC, *PA* + comorbid pain-related CHC, *NE* + Comorbid neurological CHC, *OR* Odds ratio, *CI* Confidence interval, *NA* Not applicable

^a^Odds ratios are reported for the comparison of men with vs. without CHC

^b^Odds ratios were not calculated because there was no event in one group

### Accessed and preferred information sources

**Fig. 2:**
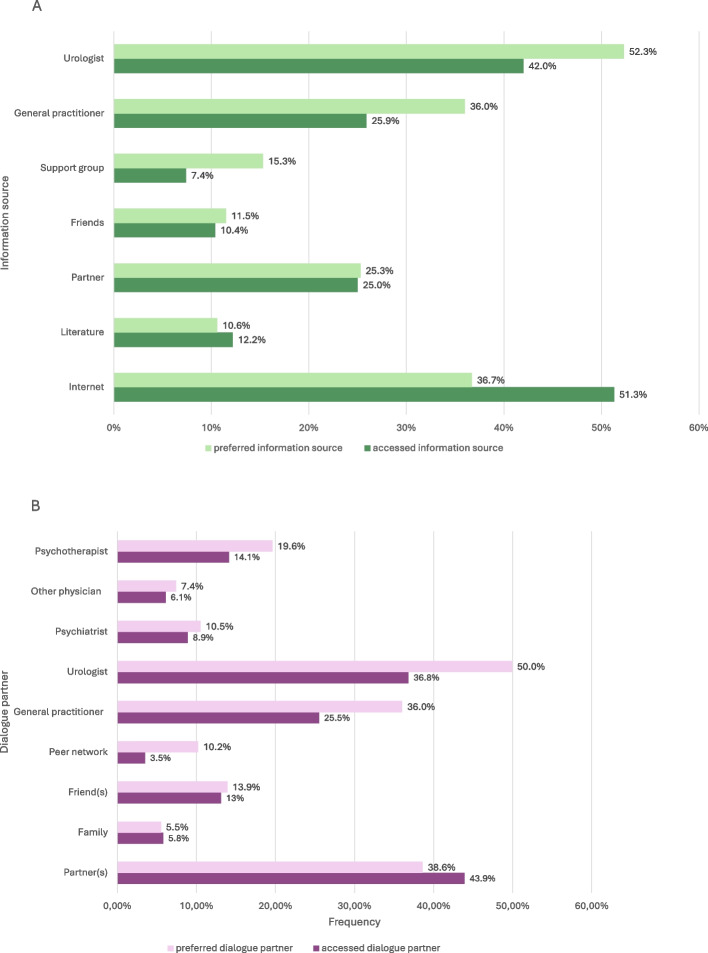
Accessed and preferred information sources and dialogue partners in all men with SD. This bar chart shows accessed versus preferred information sources and dialogue partners among men with SD. Panel **A** shows information sources; Panel **B** shows dialogue partners. Light-colored bars indicate preferred sources, and dark-colored bars indicate accessed sources. Percentages are weighted. Abbreviation: SD, sexual dysfunction

The most accessed information source among all men with SD was the internet, whereas men with CHC were significantly less likely to use the internet (45.9%) compared to men without CHC (64.5%, OR 0.47, 95% CI 0.24–0.89, see Fig. 2 (Panel A) and Table 3. Urologists were the second most named information source and were mentioned significantly more often by men with CHC compared to those without (no CHC 31.1%, CHC 46.5%; OR 1.93, 95% CI 1.00–3.88). Besides the accessed information source in the past, the top preferred sources of information were urologists (no CHC 44.6%, CHC 52.7% see Fig. 2 (Panel A) and Table 4. The exchange with their partner was reported more frequently as a preferred information source by men with MH + than by those without (MH + 31.7%, CHC MH- 21.4%).

**Table 4:** Healthcare preferences and needs of men with SD among CHC and MH status, and subgroups

	**no CHC (*n* = 69)**	**CHC (*n* = 197)**	**OR (95% CI)**^**a**^	**MH + (*n* = 87)**	**CHC MH- (*n* = 104)**	**CV + (*n* = 123)**	**UR + (*n* = 54)**	**IN + (*n* = 36)**	**CA + (*n* = 24)**	**PA + (*n* = 42)**	**NE + (*n* = 15)**
**Information sources, n (%)**	66	190		84	102	119	54	35	23	42	15
Internet	28 (43.1)	65 (34.2)	0.69 (0.39–1.23)	30 (35.1)	33 (32.8)	35 (29.3)	17 (30.7)	16 (45.3)	7 (29.2)	14 (34.4)	2 (13.2)
Literature	7 (11.1)	19 (10.1)	0.90 (0.38–2.38)	9 (10.4)	11 (10.3)	11 (9.5)	5 (9.3)	5 (13.5)	3 (11.7)	4 (9.1)	1 (6.3)
Partner(s)	16 (23.8)	49 (25.5)	1.10 (0.58–2.17)	27 (31.7)	22 (21.4)	29 (24.2)	11 (20.1)	7 (19.0)	5 (19.4)	8 (18.5)	4 (26.3)
Friend(s)	9 (13.0)	22 (11.7)	0.89 (0.39–2.19)	9 (11.1)	13 (12.7)	14 (11.7)	5 (9.6)	2 (6.2)	1 (4.5)	4 (10.0)	3 (21.2)
Peer network	15 (22.2)	25 (12.9)	0.52 (0.25–1.09)	12 (14.8)	11 (10.8)	11 (9.3)	8 (15.7)	6 (16.7)	3 (13.7)	5 (12.6)	1 (7.0)
General practitioner	18 (27.2)	72 (38.0)	1.64 (0.89–3.11)	36 (42.1)	35 (34.3)	52 (43.5)	12 (21.7)	10 (28.6)	6 (25.4)	17 (40.4)	7 (44.2)
Urologist	29 (44.6)	100 (52.7)	1.38 (0.79–2.45)	43 (51.4)	56 (54.7)	71 (59.5)	32 (59.6)	22 (62.5)	16 (68.1)	22 (52.5)	9 (60.2)
**Dialogue partners, n (%)**	63	189		83	102	118	53	34	23	41	14
Partner(s)	22 (34.7)	75 (39.7)	1.24 (0.69–2.28)	32 (38.6)	43 (42.1)	47 (39.9)	18 (33.5)	12 (34.6)	11 (46.2)	14 (33.8)	4 (26.9)
Family	6 (9.8)	9 (4.8)	0.46 (0.16–1.42)	3 (3.6)	6 (5.9)	6 (5.1)	2 (4.2)	1 (3.0)	1 (4.5)	4 (10.5)	2 (15.49
Friend(s)	8 (12.4)	26 (13.9)	1.14 (0.50–2.86)	16 (18.6)	11 (10.5)	14 (11.7)	6 (11.8)	6 (17.8)	3 (12.0)	4 (10.8)	3 (23.7)
Peer network	4 (6.0)	25 (13.2)	2.35 (0.85–8.54)	10 (12.4)	15 (14.3)	15 (12.4)	9 (16.6)	6 (17.5)	4 (17.8)	8 (18.4)	3 (22.4)
General practitioner	21 (32.7)	67 (35.6)	1.13 (0.62–2.11)	28 (33.4)	39 (37.7)	48 (41.1)	14 (26.0)	10 (30.6)	6 (24.4)	12 (30.0)	5 (35.0)
Urologist	26 (40.9)	98 (51.5)	1.54 (0.87–2.77)	39 (46.3)	57 (55.9)	68 (57.4)	29 (53.6)	20 (58.2)	14 (60.0)	22 (52.8)	6 (42.4)
Psychiatrist	4 (6.9)	22 (11.4)	1.73 (0.65–5.80)	15 (17.5)	7 (6.9)	11 (9.1)	4 (8.0)	5 (14.2)	1 (4.1)	5 (12.3)	3 (22.5)
Other physician	5 (7.8)	13 (6.6)	0.83 (0.30–2.73)	4 (4.5)	8 (7.7)	10 (8.1)	4 (7.7)	3 (7.6)	1 (4.4)	1 (2.5)	1 (6.4)
Psychotherapists	8 (13.3)	40 (20.9)	1.73 (0.80–4.13)	26 (30.8)	13 (12.6)	18 (15.1)	9 (17.0)	11 (31.6)	3 (11.5)	10 (24.4)	1 (7.6)
Police	2 (3.3)	1 (0.5)	0.16 (0.01–1.62)	1 (1.2)	0 (0)	0 (0)	1 (1.9)	0 (0)	0 (0)	0 (0)	0 (0)
Lawyer	0 (0)	6 (3.4)	NA^**b**^	1 (1.1)	6 (5.4)	3 (2.7)	5 (8.5)	0 (0)	1 (5.3)	1 (2.7)	1 (7.6)
Nobody	7 (10.3)	5 (2.7)	0.24 (0.07–0.80)								
**Treatment goals, n (%)**	62	192		86	102	118	54	35	23	42	15
Increased QoL	15 (24.1)	54 (27.9)	1.22 (0.64–2.43)	22 (26.0)	30 (29.7)	36 (30.2)	14 (25.2)	10 (27.7)	10 (44.3)	15 (36.7)	7 (46.1)
Health literacy	8 (13.0)	13 (6.8)	0.48 (0.19–1.28)	7 (8.0)	6 (5.9)	9 (7.6)	8 (15.0)	4 (10.7)	2 (8.4)	3 (7.5)	4 (26.2)
Sexual satisfaction	26 (41.6)	101 (52.8)	1.57 (0.88–2.83)	43 (50.3)	56 (55.0)	71 (60.3)	23 (42.1)	20 (57.4)	10 (43.5)	17 (40.4)	5 (30.1)
Relationship satisfaction	29 (45.9)	71 (36.9)	0.69 (0.38–1.24)	37 (42.9)	32 (31.2)	46 (38.8)	13 (23.4)	15 (41.9)	11 (48.0)	14 (32.5)	8 (50.7)
Feeling safe and close	4 (7.1)	14 (7.3)	1.04 (0.36–3.60)	9 (10.7)	5 (4.7)	7 (6.0)	5 (9.6)	1 (4.3)	1 (3.3)	5 (12.3)	4 (27.7)
Body and sexual self-esteem	18 (29.5)	56 (29.3)	0.99 (0.53–1.89)	30 (35.0)	24 (23.7)	34 (28.6)	11 (20.2)	11 (32.0)	9 (40.5)	15 (35.2)	3 (20.8)
Less stress	15 (23.5)	36 (18.7)	0.74 (0.38–1.52)	17 (19.5)	17 (16.7)	21 (17.7)	9 (16.2)	7 (20.5)	4 (15.3)	9 (20.4)	0 (0)
Sex for relaxation	13 (20.1)	46 (23.9)	1.25 (0.63–2.63)	22 (26.1)	22 (21.1)	33 (27.7)	17 (30.6)	9 (27.0)	7 (31.0)	12 (29.3)	6 (38.8)
Increased desire	15 (24.6)	63 (33.1)	1.52 (0.81–3.00)	31 (36.1)	31 (30.0)	40 (33.7)	15 (27.8)	10 (29.7)	5 (23.2)	18 (43.9)	6 (39.8)
Increased arousal	20 (31.5)	64 (33.6)	1.10 (0.60–2.07)	28 (33.1)	36 (35.3)	43 (36.8)	20 (36.2)	13 (37.6)	7 (29.0)	17 (40.6)	3 (19.4)
Frequent orgasm	9 (14.4)	37 (19.2)	1.42 (0.66–3.34)	19 (22.0)	18 (17.7)	23 (19.4)	12 (21.5)	8 (23.9)	4 (16.0)	8 (19.5)	1 (6.5)
Decreased pain	2 (3.4)	17 (8.6)	2.64 (0.74–15.95)	11 (13.3)	5 (5.0)	10 (8.5)	7 (13.8)	6 (16.6)	2 (9.5)	3 (7.1)	0 (0)
More communication	6 (10.1)	11 (5.8)	0.55 (0.20–1.62)	6 (6.9)	4 (4.1)	6 (4.9)	4 (7.7)	2 (4.8)	2 (7.7)	4 (9.7)	0 (0)
Increased knowledge	4 (7.0)	12 (6.2)	0.88 (0.30–3.12)	5 (6.1)	7 (6.6)	8 (6.5)	6 (10.7)	2 (4.8)	3 (12.0)	2 (4.4)	0 (0)
Social participation	1 (1.3)	7 (3.5)	2.69 (0.41–79.58)	4 (4.5)	3 (2.7)	3 (2.5)	3 (5.2)	1 (3.1)	2 (8.6)	1 (2.4)	1 (7.0)
**Preferred offerings, n (%)**	69	197		87	104	123	54	36	24	42	15
Specialized clinics	12 (17.3)	38 (19.2)	1.14 (0.56–2.43)	17 (20.0)	20 (19.6)	24 (19.8)	11 (20.1)	12 (34.3)	4 (16.2)	11 (25.8)	1 (7.0)
Drugs	25 (36.7)	84 (42.6)	1.28 (0.73–2.29)	35 (40.2)	46 (44.3)	57 (46.4)	19 (34.3)	16 (44.8)	10 (41.2)	22 (51.4)	5 (33.0)
Surgery	3 (4.2)	17 (8.8)	2.18 (0.69–9.85)	7 (8.0)	9 (8.9)	12 (9.6)	8 (15.6)	3 (8.0)	2 (9.3)	7 (16.7)	3 (21.1)
Psychotherapy	14 (20.2)	32 (16.3)	0.77 (0.39–1.59)	20 (23.4)	12 (11.2)	19 (15.2)	7 (12.7)	8 (21.6)	3 (11.8)	7 (16.2)	0 (0)
Sex and couples therapy	15 (22.3)	30 (15.0)	0.62 (0.31–1.26)	16 (18.5)	14 (13.0)	15 (12.6)	10 (19.2)	12 (33.5)	4 (15.9)	7 (15.6)	4 (27.2)
Relaxation methods	20 (29.3)	40 (20.3)	0.61 (0.33–1.16)	18 (21.3)	21 (19.7)	18 (14.5)	11 (20.6)	9 (24.4)	3 (11.5)	9 (22.4)	3 (21.0)
Peer counseling	9 (12.9)	28 (14.2)	1.11 (0.51–2.65)	16 (18.9)	11 (11.0)	19 (15.4)	8 (14.8)	7 (18.3)	4 (15.1)	5 (11.7)	2 (13.3)
Physiotherapy	10 (13.8)	24 (12.1)	0.86 (0.39–2.02)	14 (15.9)	10 (9.6)	11 (8.9)	7 (13.8)	3 (8.6)	3 (13.2)	7 (17.6)	2 (14.3)
Pelvic floor training aids	9 (12.5)	17 (8.4)	0.64 (0.27–1.62)	6 (7.0)	11 (10.1)	9 (7.6)	7 (12.8)	5 (12.8)	3 (12.1)	4 (9.5)	0 (0)
Biofeedback	4 (6.5)	11 (5.6)	0.85 (0.29–3.01)	7 (8.3)	3 (2.8)	5 (4.0)	4 (7.3)	3 (7,6)	1 (3.2)	3 (6.9)	2 (13.8)
Penispump	5 (7.4)	19 (9.7)	1.35 (0.52–4.20)	11 (12.1)	9 (8.3)	14 (11.7)	6 (11.6)	3 (8.4)	2 (7.9)	4 (10.0)	3 (20.7)
Body therapies (e.g., osteopathy massage)	12 (17.9)	25 (12.7)	0.67 (0.32–1.46)	8 (9.1)	16 (15.6)	13 (10.2)	10 (17.6)	6 (15.8)	6 (25.7)	7 (16.8)	1 (7.2)
Physical activity	19 (27.5)	38 (19.4)	0.63 (0.34–1.22)	17 (19.7)	19 (18.3)	22 (18.1)	10 (19.4)	5 (12.7)	5 (20.0)	7 (16.7)	4 (28.1)
Nutrition	6 (9.4)	21 (10.9)	1.18 (0.49–3.25)	11 (13.1)	9 (8.6)	11 (9.2)	5 (9.1)	3 (7.5)	2 (7.5)	6 (14.2)	2 (13.9)
**Favored future developments, n (%)**	64	180		80	96	110	53	34	22	42	14
Drugs	22 (35.2)	80 (44.6)	1.48 (0.82–2.72)	29 (35.9)	50 (51.7)	53 (47.9)	17 (32.1)	14 (39.5)	8 (38.3)	19 (44.7)	4 (26.0)
Surgery	4 (6.5)	22 (12.2)	2.02 (0.74–7.07)	11 (13.4)	11 (11.7)	18 (16.2)	7 (13.3)	4 (12.1)	5 (23.8)	7 (15.9)	4 (30.1)
Information offerings	21 (32.6)	37 (20.8)	0.54 (0.29–1.04)	22 (27.2)	15 (15.3)	19 (17.5)	6 (10.5)	13 (39.0)	3 (15.8)	7 (17.8)	2 (11.9)
Digital offers											
*app*	14 (21.4)	25 (13.8)	0.59 (0.28–1.26)	14 (17.9)	9 (8.9)	14 (12.7)	6 (11.7)	6 (18.6)	3 (14.1)	5 (12.0)	2 (16.0)
*website*	13 (19.9)	27 (14.7)	0.70 (0.33–1.51)	12 (15.3)	14 (14.9)	16 (14.2)	6 (11.1)	2 (5.2)	4 (17.1)	4 (9.8)	3 (22.0)
*home-aids*	9 (14.2)	33 (18.6)	1.38 (0.64–3.26)	12 (14.7)	22 (22.6)	23 (21.3)	19 (36.0)	7 (21.7)	5 (23.0)	11 (25.3)	1 (7.5)
*with physical face-to-face treatments*	5 (7.9)	16 (9.1)	1.16 (0.43–3.68)	9 (11.0)	8 (7.9)	9 (8.6)	7 (13.4)	7 (19.3)	1 (4.7)	5 (11.8)	2 (14.7)
*contact to experts*	9 (14.2)	22 (12.2)	0.84 (0.37–2.02)	12 (15.1)	10 (10.3)	14 (12.8)	4 (7.6)	9 (25.0)	3 (13.7)	5 (12.3)	2 (14.8)
Trainings											
*of physicians*	5 (8.0)	32 (18.1)	2.53 (1.02–7.69)	14 (17.9)	17 (18.0)	21 (19.2)	12 (21.7)	6 (16.2)	8 (38.8)	8 (18.3)	4 (27.2)
*of psychologists*	16 (25.8)	28 (15.8)	0.54 (0.27–1.10)	14 (17.6)	14 (14.9)	18 (16.0)	9 (17.6)	6 (17.0)	5 (24.5)	7 (16.3)	3 (20.7)
*diversity and trauma*^*b*^	8 (12.1)	18 (10.0)	0.81 (0.34–2.12)	10 (12.3)	8 (8.5)	8 (6.9)	7 (13.9)	2 (4.9)	2 (7.8)	5 (12.2)	1 (8.0)
**Design of digital offers**^**c**^**, n (%), Median (IQR)**	60	181		81	96	108	53	32	22	39	14
Stand-alone	6.0 (4.4–9.0)	7.0 (5.0–8.0)		7.0 (5.0—8.0)	7.0 (5.0–8.0)	7.0 (5.0—9.0)	7.0 (6.0—9.0)	7.0 (5.0—8.0)	8.0 (6.4—9.1)	6.6 (5.0—8.0)	7.0 (6.4—9.0)
Possibility to contact experts	7.0 (5.0—9.0)	8.0 (6.0—10.0)		8.0 (7.0—10.0)	8.0 (5.3—10.0)	8.0 (7.0—10.0)	8.0 (6.1—10.0)	8.0 (7.0—10.0)	8.0 (7.0—9.0)	9.0 (6.8—10.0)	8.0 (7.0—10.0)
Integration of medical HCP	7.0 (5.0–8.0)	7.0 (5.0–9.0)		7.0 (6.0—9.0)	7.0 (5.0—9.0)	8.0 (6.0—9.5)	8.0 (5.0—9.0)	7.0 (5.7—9.2)	7.7 (5.0—9.0)	7.1 (5.0—9.0)	8.0 (7.0—10.0)
Integration of psychological HCP	7.0 (5.0–8.0)	7.0 (5.0–8.0)		7.0 (5.0—8.0)	6.6 (5.0—8.0)	7.0 (5.0—9.0)	7.1 (5.0—8.0)	7.0 (5.0—8.3)	7.7 (5.0—8.0)	7.0 (5.0—8.0)	8.0 (6.4—10.0)
Possibility to incorporate partners	7.0 (5.0—9.0)	7.0 (5.0—9.0)		7.0 (4.5—9.0)	7.0 (5.0—9.0)	7.0 (4.6—9.0)	7.0 (5.0—9.0)	7.0 (4.1—10.0)	7.2 (5.6—9.0)	8.0 (4.8–10)	8.0 (5.7—9.1)
Reimbursement	9.0 (6.0—10.0)	9.0 (7.0—10.0)		10.0 (7.8—10.0)	9.0 (6.0—10.0)	9.0 (7.0—10.0)	10.0 (8.0—10.0)	9.0 (7.0—10.0)	9.7 (6.4—10.0)	10.0 (8.0—10.0)	9.2 (8.0—10.0)
**Expert contact, n (%)**	61	170		77	91	103	51	32	20	38	13
Chat	20 (33.4)	48 (28.3)	0.79 (0.42–1.50)	23 (30.2)	25 (27.6)	29 (28.5)	17 (33.8)	11 (34.9)	8 (38.5)	15 (39.6)	6 (45.0)
Video call	13 (20.6)	30 (17.8)	0.83 (0.40–1.79)	10 (13.5)	19 (20.8)	12 (11.7)	12 (22.9)	9 (27.9)	6 (30.9)	8 (21.2)	3 (25.6)
E-Mail Feedback	19 (30.9)	49 (28.9)	0.91 (0.48–1.75)	25 (32.5)	22 (24.7)	29 (28.6)	13 (25.7)	7 (22.5)	7 (33.3)	12 (32.3)	4 (32.2)
Contact to medical sex experts	24 (39.1)	69 (40.4)	1.05 (0.58–1.94)	35 (45.7)	34 (37.2)	38 (36.9)	20 (38.2)	17 (51.4)	9 (46.4)	19 (49.7)	6 (44.6)
Contact to psychological sex experts	27 (44.4)	79 (46.2)	1.08 (0.60–1.96)	43 (56.5)	34 (38.0)	48 (47.2)	21 (41.4)	16 (50.0)	6 (28.2)	17 (43.8)	8 (60.6)
**Amount willing to pay, n (%)**	29	82		37	43	50	25	17	10	19	8
Nothing	1 (4.3)	6 (7.3)		3 (8.1)	3 (6.9)	2 (3.9)	0 (0)	0 (0)	1 (9.9)	0 (0)	0 (0)
< €50	6 (20.0)	22 (27.3)		7 (19.6)	13 (30.6)	16 (32.5)	6 (25.2)	1 (6.3)	3 (31.3)	6 (31.8)	3 (38.9)
€51–100	7 (23.0)	20 (24.3)		12 (33.0)	8 (17.9)	11 (22.0)	5 (19.6)	9 (54.6)	1 (8.5)	7 (34.9)	1 (12.6)
€101–300	4 (15.1)	9 (10.8)		6 (15.4)	3 (7.4)	5 (10.0)	4 (17.4)	1 (4.7)	1 (7.6)	3 (13.2)	0 (0)
> €300	11 (37.6)	25 (30.3)		9 (23.8)	16 (37.2)	16 (31.6)	9 (37.8)	6 (34.4)	4 (42.7)	4 (20.0)	4 (48.5)

This table presents detailed findings on the healthcare preferences and needs of men with sexual dysfunction (SD), stratified by overall chronic health condition (CHC) and mental health-related CHC status, and comorbid CHC subgroups. Variables include information sources, dialogue partners, treatment goals, preferred offerings, favored future developments, design of digital offers, expert contact, and amounts willing to pay. Categorical variables are shown as weighted frequencies; ordinal variables are presented as weighted medians with interquartile ranges (IQR). Odds ratios (OR) with 95% confidence intervals (CI) are reported for the comparison of men with versus without CHC, calculated using weighted logistic regression

*Abbreviation*: *SD* Sexual dysfunction, *CHC* Chronic health conditions, *MH* + Comorbid mental CHC, *CHC MH-* CHC excluding MH, *CV* + Comorbid cardiovascular and metabolic CHC, *UR* + Comorbid urological CHC, *IN* + Comorbid infectious and inflammatory CHC, *CA* + Comorbid cancer CHC, *PA* + comorbid pain-related CHC, *NE* + Comorbid neurological CHC, *HCP* Healthcare provider, *OR* Odds ratio, *CI* Confidence interval, *IQR* Interquartile ranges

^a^Odds ratios are reported for the comparison of men with vs. without CHC

^b^Sensitivity trainings for e.g., culture, religion, trauma, gender identity or sexual orientation

^c^Numeric rating scale from 1–10 (1 = not important to 10 = very important)

### Accessed and preferred dialogue partners

As previous dialogue partners for sexual health concerns, partners were most frequently reported (no CHC 47.6%, CHC 40.9%), followed by urologists (no CHC 24.7%, CHC 41.4%; see Fig. 2, Panel B and Table 3. Men with CHC contacted urologists significantly more often in the past than men without CHC to talk about sexual problems (OR 2.15, 95% CI 1.07–4.59). Most preferred dialogue partners were urologists regardless of CHC status (no CHC 40.9%, CHC 51.5%). Men with MH + more often expressed a preference to consult psychiatrists (MH + 17.5%, CHC MH- 6.9%) and psychotherapists (MH + 30.8%, CHC MH- 12,6%) as dialogue partners than men without MH + (see Fig. 2, Panel B and Table 4. Further information on specific subgroups can be found in Tables 3 and 4.

### Need for therapy

Among the used treatment offers in the past, Urological examinations were frequently used by men with SD, with higher rates among men with CHC (25.8%) compared to those without CHC (11.1%, OR 2.78, 95% CI 1.12–8.38). Rates were similar in men with MH + (26.2%) and CHC MH– (25.3%). Medication was the most frequently offered treatment across all groups (no CHC 28.4%, CHC 42.5%, MH + 37.0%, CHC MH- 47.9%). It was reported approximately twice as often by men with CHC compared to those without (OR 2.08, 95% CI 1.05–4.35). Other frequently accessed therapeutic recommendations were physical activity (no CHC 24.2%, CHC 14.6%, MH + 19.9%, CHC MH + 9.0%), diet (no CHC 6.0%, CHC 14.4%, MH + 15.7%, CHC MH- 12.5%) and the use of a penis pump (no CHC 6.7%, CHC 12.0%, MH + 18.3%, CHC MH- 5.5%). Psychotherapy was more frequently reported as an offered treatment for sexual problems among men with MH + compared to those without (MH + 12.9%, CHC MH- 1.2%). Use of sex or couple’s therapy was low in both groups (no CHC 6.7%, CHC 3.9%).

When asked for their preferred treatment offerings, men across all groups considered medication as their most preferred therapy (no CHC 36,7%, CHC 42.6%, MH + 40.2%, CHC MH- 44.3%). Relaxation methods (no CHC 29.3%, CHC 20.3%, MH + 21.3%, CHC MH- 19.7%), specialized clinics (no CHC 17.3%, CHC 19.2%, MH + 20.0%, CHC MH- 19.6%) and physical activity (no CHC 27.5%, CHC 19.4%, MH + 19.7%, CHC MH- 18.3%) were further mentioned in most groups. Sex and couples therapy was desired by 22.3% of men with no CHC and 15.0% with CHC. Psychotherapy as preferred treatment offer was reported twice as often by men with MH + compared to those without (MH + 23.4%, CHC MH- 11.2%). Similarly, preferences for sex and couples therapy was more often reported my men with MH + (MH + 18.5%, CHC MH- 13.0%). More information about accessed and preferred treatment across CHC subgroups can be found in Tables 3 and 4.

### Treatment motivation

Regarding motivation and subjective goals for therapy, the most prioritized aspects reported were relationship satisfaction (no CHC 45.9%, CHC 36,9%, MH + 42.9%, CHC MH- 31.2%), and sexual satisfaction (no CHC 41.6%, CHC 52,8%, MH + 50.3%, CHC MH- 55.0%). Functional improvements, such as increased desire (no CHC 24.6%, CHC 33.1%, MH + 36.1%, CHC MH- 30.0%) and sexual arousal (no CHC 31.5%, CHC 33.6%, MH + 33.1%, CHC MH- 35.3%), were also frequently mentioned by men with SD. The median willingness to pay for effective help was €101–300 among men without CHC, and €51–100 among those with CHC, regardless of mental health status. More information about treatment goals and willingness to pay across CHC subgroups can be found in Table 4.

### Need for future developments and digital interventions

The most anticipated future development was the introduction of new medication (no CHC 35.2%, CHC 44.6%). This development was rated as more important by men without MH + than by men with MH + (MH + 35,9%, CHC MH- 51,7%). The second most desired development was better information offerings (no CHC 32.6%, CHC 20.8%). In contrast to medication, this development was considered more important by men with MH + than by men without MH + (MH + 27.2, CHC MH- 15.3%). Regarding the specialization of different health care providers, trainings for psychologists were more important to men without CHC (no CHC 25.8%, CHC 15.8%, OR 0.54, 95%, CI 0.27–1.10), whereas trainings for physicians were more often reported to important by men with CHC (no CHC 8.0%, CHC 18.1%, OR 2.53, 95% CI 1.02–7.69).

When asking for preferred digital offers, men without CHC more often wished for an app (no CHC 21.4%, CHC 13,8%) or a website with information and exercises (no CHC 19.9%, CHC 14.7%), whereas men with CHC preferred home aids coming along digital offers (no CHC 14.2%, CHC 18.6%). For all groups, the possibility of reimbursement was the most important aspect of a digital offer, see Table 4.

## Discussion

The present study aimed to examine the prevalence of self-reported clinical diagnosis of SD, help-seeking behavior and healthcare needs related to male SD, focusing on differences in physical and mental CHC. Overall, only 46.6% of men meeting the ICD-11 SD criteria reported having received a diagnosis in the healthcare system. The gap between symptom reporting and diagnosis was most pronounced for men without CHC (32.6% vs. 50.8% in CHC), while men with mental CHC were the most likely to receive a diagnosis (53.3% vs. 48.7% in CHC MH-). The diagnostic gap between the presence of SD symptoms and received diagnoses was most pronounced between men with MH + and those without any CHC (53.3% vs. 32.6%; OR 2.36, 95% CI 1.15–4.84). Regarding preferred sources of information, urologists were the most favored, followed by the internet. Urologists remained a key source of support for both groups, though men with MH + considered psychotherapists and psychiatrists more important than those without. Accordingly, having accessed psychotherapy for SD-related issues was reported more often by men with MH + were than men without MH +. Therapy initiation rates were low across all groups, with a median waiting time of 3–4 months for treatment. Men reported medication as the most commonly used treatment, the most desired therapeutic option, and the area most in need of future development. In addition to pharmacological interventions, relaxation techniques and physical activity were also identified as preferred non-medication-based therapy options. Although sex and couples therapy was a commonly desired option, it had not been widely accessed in the past. In terms of digital health services, men without CHC favored apps or websites with information and exercises, while men with CHC preferred home aids to complement digital solutions. Regarding treatment goals, sexual satisfaction was prioritized by men with CHC, while relationship satisfaction was more important to men without CHC. Reimbursement was a key concern for all, though men without CHC were more willing to pay for services. Shame was identified as the main barrier to help-seeking, particularly among men with MH +.

### Needs and preferences

Our results show the internet to be the most used information source, while urologists were the preferred source of information. This finding is consistent with previous studies highlighting the discrepancy between accessibility and perceived credibility of information sources in the context of sexual health [32, 33, 37, 38]. The increased use of the internet to acquire information on sexuality may be attributed to its around-the-clock accessibility, ease of use, and the perceived anonymity it offers, which may reduce barriers to seeking information on sensitive topics [48]. Partners were found to be the most reported contact person, an observation that aligns with other research [32, 37] and supports that being in a relationship can serve as a protective factor for SD [49], also in patients with CHC, as presented in the unpublished work of Kronthaler et al. on men. Men with CHC were more likely to consult urologists as dialogue partners than men without CHC, while men with mental health conditions more frequently contacted mental health professionals than those without. These increased interactions with health professionals may result from regular visits for other health reasons such as routine check-ups, medication monitoring, or therapy sessions for their somatic or mental comorbid condition [50, 51], potentially creating opportunities to address SD in familiar care settings. This finding highlights the importance of established, trusted physician–patient relationships for effective self-management [52], while also underscoring the need to improve access and health literacy for individuals with limited healthcare contact [53]. In our study, only 13,6% of men with SD and without CHC and 15.8% of men with SD and CHC received treatment. These treatment rates were lower than in most other international cross-sectional studies, in which treatment rates range from 9 to 43% [11, 32, 33, 35, 36]. The discrepancy in observed treatment rates between studies may reflect the lack of population-based data, differences in sample characteristics (e.g., age, comorbidities), and international variations in help-seeking. Despite meeting diagnostic criteria, over 50% of men with SD in this study had not received a formal diagnosis within the healthcare system. This observation supports prior findings on the underrecognition of male sexual concerns [30, 31] and highlights emerging barriers to help-seeking, such as shame and limited availability of services, as observed in our study. In addition, some sexual issues may be generally well tolerated and cause little distress, for example in individuals who are not sexually active and do not wish to be. Men with CHC were twice as likely to have received an SD diagnosis compared to those without CHC, suggesting that more frequent medical consultations in this group [50, 51] may offer increased opportunities to address sexual health. The percentage of received SD diagnosis was similar in men with somatic and mental CHC. ED was the most frequently diagnosed condition across all subgroups, reflecting both the extensive body of research on ED and the comparatively high availability and utilization of pharmaceutical treatments for this condition. Notably, men with CHC were 4.55 times more likely to be diagnosed with HSDD than men without CHC. This suggests that desire-related concerns are more often detected in the context of broader medical consultations — for example, when HSDD emerges as a side effect of CHC treatment — rather than presented as a primary reason for seeking help. In men with mental health conditions, PE was more frequently diagnosed (18.0%) than in those without (9.2%), while rates of other SD diagnoses were comparable across groups. This finding suggests that PE might be more often conceptualized as a mental health–related dysfunction, whereas ED tends to be more strongly associated with cardiovascular and other somatic conditions, as suggested by the unpublished findings of Kronthaler et al. on men. Nevertheless, both conditions have a biopsychosocial etiology which might be underscored in different directions for distinct SD domains.

### Barriers

In light of the persistently low rates of help-seeking and the diagnostic gaps, the present study identified shame as the most significant perceived barrier to actively seek professional support. This finding matched other studies reporting embarrassment to be a major barrier to help seeking in men with SD [32, 33, 36–38]. The stigmatization and taboo around sexual issues may be rooted in cultural norms, societal expectations, or a lack of open dialogue and may discourage individuals from directly consulting healthcare professionals [54]. Our findings show that shame may be particularly present in men also affected by mental health conditions, compared to men with somatic CHC only. Men experiencing mental health problems may frequently experience significant self-stigma, which may foster shame and function as a barrier in help-seeking [55]. In addition, the perception of a dysfunction as psychogenic rather than biologically caused may be associated with stronger feelings of shame. Destigmatizing sexual issues may further normalize the topic in healthcare settings and encourage earlier, more proactive help-seeking [56]. Notably, men with mental CHCs not only reported greater access to psychosexual therapy but also more frequently expressed the need for psychologist-targeted training compared to men without MH +. This difference may reflect heightened awareness of the psychosocial determinants of SD, fostered through psychotherapy and associated psychoeducation [55]. Additionally, positive experiences in overcoming stigma and seeking professional help in the past may facilitate future help-seeking behavior [57–59]. A deeper understanding of the biopsychosocial etiology of illness, together with prior engagement in psychosocial therapies, may also enhance openness to psychosocial interventions addressing sexuality.

### Access to treatment modalities

The most reported therapy for treating SD was medication use. This high prevalence of medication use may be attributed to the rapid onset and ease of use of pharmacological agents, such as phosphodiesterase-5 inhibitors for ED [60]. Reported rates of medication use for SD varied considerably across previous studies [2, 11, 12, 32–34]. Respective differences may be due to different characteristics of study populations such as age and the presence of certain comorbidities, but also to differing national treatment guidelines or access to treatment in various countries.

Furthermore, men in this study most frequently reported medication as the most desired future treatment for SD. This preference may reflect structural conditions of the healthcare system, where pharmaceutical treatment plays a dominant role. According to OECD data, Germany ranks among the countries with the highest levels of pharmaceutical consumption, particularly in primary care and chronic disease management [61]. Such emphasis on medication-based approaches may contribute to men being less aware of, or less likely to seek, alternative non-pharmacological interventions.

Moreover, the understanding of non-medical treatment options for male SD other than ED remains limited: To date, psychological intervention studies for male SD, such as HSDD, OD or dyspareunia in men, remains scarce [28]. This striking research gap may constrain evidence-based treatment development and clinical recommendations for these conditions.

### Potential and challenge of digital interventions

Given the limited availability of reimbursable non-pharmacological sexual therapies in Germany, it is notable that interest in digital interventions was low: fewer than one quarter of men expressed such interest, with particularly low rates among those with CHC. This finding underscores a significant gap between the existing need for accessible sexual health interventions and the current appeal or perceived suitability of digital solutions among male patients. Furthermore, high dropout rates in digital psychological interventions [29, 41] suggest that these approaches may not fully address patients' needs. Platforms that offer combined options for integration of human interaction, such as guided support, live chat with experts, or periodic feedback, might be better suited to meet user preferences and enhance adherence [62–66].

### Implications for clinical health care and research

This study revealed differences in healthcare needs and preferences among distinct subgroups. These findings highlight the necessity of individualized treatment strategies to adequately meet the specific needs of patients with SD. The gap between actual and preferred information sources highlights the need to provide easily accessible, low-threshold consultation options [67] with sexologist or sexual medicine specialists within the healthcare system, as comprehensive knowledge is crucial. For instance, a urologist with training in sexual medicine may recognize treatment-related side effects, such as decreased libido with paroxetine for premature ejaculation, while a sexual medicine-trained psychiatrist can order tests to rule out organic causes. In particular, certain rare conditions, such as pudendal neuralgia, pudendal or spinal nerve injury, require specific diagnostic testing, including neurological exams, magnetic resonance imaging, genitourinary perineal electroneuromyography, or somatosensory evoked potential [68]. Greater professional knowledge of sexual dysfunctions, their triggers, and correlates thus enhances the ability to resolve sexual issues.

Established continuous physician–patient relationships should be leveraged to enhance patients' self-management [52]. Healthcare professionals must also address perceived shame as a barrier to care by fostering a nonjudgmental environment and proactively initiating discussions about sexual wellbeing during routine visits [54]. The role of stigma and sexual taboos in Germany warrants further investigation, as reducing such barriers may facilitate help-seeking behavior [69]; accordingly, future research should aim to develop and evaluate targeted interventions to address these psychosocial obstacles.

The offered personalized treatment should focus on biopsychosocial factors, address all dimensions of sexuality [70]. This may include psychological interventions such as cognitive behavioral therapy and educational support, as well as pharmacological or technology-based approaches also allowing for partner inclusion [71]. Partner-inclusive approaches can strengthen treatment outcomes and relationship dynamics [72]. In Germany, the reimbursement of couple-based programs underscores the relevance of integrating relational aspects into sexual healthcare and contributes to better accessibility of these interventions.

Given the existing treatment gap, a consultation – also delivered online – with a sexologist or sexual medicine specialist may help overcome barriers such as embarrassment and guide the way to the necessary diagnostic tests requiring in-person assessment with physical examinations by healthcare providers other disciplines. In addition, fully digitally delivered psychosexual interventions and couple therapies are emerging as structured, modular solutions to address SD in the future [29, 39, 40]. Future research should investigate how these digital solutions can be better tailored to male patients' preferences, particularly by exploring the impact of integrating experts' interaction on user engagement, satisfaction, and adherence. In this context, patient involvement and qualitative methods should be considered. Additionally, understanding barriers to the acceptance of digital psychosocial formats could inform the development of more appealing and effective hybrid therapeutic models. Establishing reimbursement in the German healthcare system is crucial to reduce financial barriers, especially for men with CHC, who often have limited resources [73].

### Strengths of the current study

A key strength of this study is its novelty. As to the best of our knowledge this is the first study investigating help-seeking behavior and treatment needs in men with SD in relation to the presence of CHC and across different CHC subgroups in a German population-based sample. Existing studies have primarily focused on men with specific individual conditions, such as prostate cancer or diabetes, rather than exploring broader patterns of healthcare needs and behaviors across multiple CHC [11, 37]. Furthermore, most existing comparative studies have concentrated on a single form of SD, most commonly ED [2, 11, 12, 34, 38] or, to a lesser extent, PE [33], which limits the applicability of their findings to the broader range of SD. Based on insights from this study regarding men's healthcare preferences, the findings can inform the development of tailored interventions — such as digital formats or partner-inclusive approaches — which should subsequently be evaluated for their effectiveness and potential for integration into existing healthcare systems.

### Study limitations

The study had several limitations. The sample was population-based only with respect to age, gender, and federal state, which limits the generalizability of the findings. Differences related to other characteristics may not have been adequately captured. Furthermore, the study design itself introduces several potential biases. First, the reliance on subjective self-report measures may have resulted in response or recall bias, highlighting the need for future research to incorporate complementary objective assessments. Second, the use of predefined multiple-choice formats may have introduced selection bias by constraining responses and, due to the extensive range of options, potentially leading participants to overlook relevant or nuanced experiences. Third, only German-speaking men were included impacting generalizability of the data. Additionally notable demographic differences were found between men without and with CHC in this study: Men with CHC were on average older, had less years of education and was less likely to be employed. Given the demographic disparities, future studies should also explore how social determinants of health intersect with SD and help-seeking behavior. Additionally, data on SD were missing for 9.5% of male participants, and information on received diagnosis of SD was absent in 4.6% of cases, potentially limiting the completeness and accuracy of the reported prevalence rates. Finally, our cross-sectional study design does not allow for conclusions regarding causal relationships between CHC, SD, and related psychological outcomes, which should be considered in the interpretation of our findings.

## Conclusion

This study reveals a substantial gap between men meeting ICD-11 criteria for SD and those formally diagnosed in the health care system. Observed differences in help-seeking behaviors and healthcare needs across various chronic conditions and SD underscore the importance of patient-tailored strategies addressing not only sexual function but also broader aspects of sexual health, such as relationship satisfaction. Novel digital solutions and blended care interventions, supported by reimbursement policies, could help bridge treatment gaps and improve access to specialized care. Overall, these findings highlight the unmet need for targeted efforts to enhance detection, support, and treatment for men with SD.

## Supplementary Information

Additional file 1. STROBE Statement — Checklist of items that should be included in reports of cross-sectional studies

## Data Availability

The data analyzed in this study cannot be shared publicly due to ethical restrictions.

## Abbreviations

CHC: Chronic health conditions; men with chronic health conditions
CHC MH-: Men with only somatic conditions
CV +: Men with chronic cardiovascular and metabolic conditions
ED: Erectile dysfunction
HSDD: Hypoactive sexual desire
IN +: Men with chronic infectious and inflammatory conditions
MH +: Men with chronic mental health conditions
NE +: Men with chronic neurological conditions
No CHC: Men without chronic health conditions
OD: Orgasmic dysfunction
PA +: Men with chronic pain conditions
PE: Premature ejaculation
SD: Sexual dysfunction
UR +: Men with chronic urologic conditions

## References

[1] World Health Organization. Defining Sexual Health: Report of a technical consultation on sexual health. Geneva: *WHO*; 2002. Available from: https://www3.paho.org/hq/dmdocuments/2009/defining_sexual_health.pdf

[2] Rosen RO, Fisher WA, Eardley I, Niederberger C, Nadel A, Sand M. The multinational Men’s Attitudes to Life Events and Sexuality (MALES) study: I. Prevalence of erectile dysfunction and related health concerns in the general population. Curr Med Res Opin. 2004;20(5):607–17. 10.1185/030079904125003467. 15171225 10.1185/030079904125003467

[3] Laumann EO, Paik A, Rosen RC. Sexual dysfunction in the United States: prevalence and predictors. JAMA. 1999;281(6):537–44. 10.1001/jama.281.6.537. 10022110 10.1001/jama.281.6.537

[4] Khemiri BN, Fadhel SB, Hakiri A, Homri W, Labbane R. Sexual dysfunction in the elderly: prevalence and impact on quality of life. Tunis Med. 2020;98(12):1011–6. Available from: https://latunisiemedicale.com/index.php/tunismed/article/view/3818 33480005

[5] Vasconcelos P, Carrito ML, Quinta-Gomes AL, et al. Associations between sexual health and well-being: a systematic review. Bull World Health Organ. 2024;102(12):873–87. 10.2471/BLT.24.291565. 39611198 10.2471/BLT.24.291565 PMC11601183

[6] Atlantis E, Sullivan T. Bidirectional association between depression and sexual dysfunction: a systematic review and meta-analysis. J Sex Med. 2012;9(6):1497–507. 10.1111/j.1743-6109.2012.02709.x. 22462756 10.1111/j.1743-6109.2012.02709.x

[7] Briken P, Matthiesen S, Pietras L, Wiessner C, Klein V, Reed GM, et al. Prävalenzschätzungen sexueller Dysfunktionen anhand der neuen ICD-11-Leitlinien. Dtsch Arztebl Int. 2020;117(39):653–8. 10.3238/arztebl.2020.0653. 33357346 10.3238/arztebl.2020.0653 PMC7829447

[8] Ziapour A, Kazeminia M, Rouzbahani M, Bakhshi S, Montazeri N, Yıldırım M, et al. Global prevalence of sexual dysfunction in cardiovascular patients: a systematic review and meta-analysis. Syst Rev. 2024;13(1):136. 10.1186/s13643-024-02525-0. 38769586 10.1186/s13643-024-02525-0 PMC11103881

[9] Burchardt M, Burchardt T, Baer L, Kiss AJ, Pawar RV, Shabsigh A, et al. Hypertension is associated with severe erectile dysfunction. J Urol. 2000;164(4):1188–91. 10.1016/S0022-5347(05)67138-8. 10992363

[10] Kao C-C, Lin C-L, Huang W-Y, Cha T-L, Lin T-Y, Shen C-H, et al. Association between inflammatory bowel disease and erectile dysfunction: a nationwide population-based study. Inflamm Bowel Dis. 2016;22(5):1065–70. 10.1097/MIB.0000000000000695. 26863266 10.1097/MIB.0000000000000695

[11] Rosen RC, Wing RR, Schneider S, Wadden TA, Foster GD, West DS, et al. Erectile dysfunction in type 2 diabetic men: relationship to exercise fitness and cardiovascular risk factors in the look ahead trial. J Sex Med. 2009;6(5):1414–22. 10.1111/j.1743-6109.2008.01209.x. 19192106 10.1111/j.1743-6109.2008.01209.x PMC4951185

[12] May M, Gralla O, Knoll N, Fenske S, Spivak I, Rönnebeck C, et al. Erectile dysfunction, discrepancy between high prevalence and low utilization of treatment options: results from the “Cottbus Survey” with 10 000 men. BJU Int. 2007;100(5):1110–5. 10.1111/j.1464-410X.2007.07101.x. 17922788 10.1111/j.1464-410X.2007.07101.x

[13] Leemans C, Van den Broucke S, Jeitani C. Sexual dysfunction in patients with chronic non-genital physical disease: an umbrella review. Int J Environ Res Public Health. 2025;22(2):157. 10.3390/ijerph22020157. 40003383 10.3390/ijerph22020157 PMC11855788

[14] McCabe MP, Sharlip ID, Lewis R, Atalla E, Balon R, Fisher AD, et al. Risk factors for sexual dysfunction among women and men: a consensus statement from the fourth international consultation on sexual medicine 2015. J Sex Med. 2016;13(2):153–67. 10.1016/j.jsxm.2015.12.015. 26953830 10.1016/j.jsxm.2015.12.015

[15] Jackson G, Boon N, Eardley I, Kirby M, Dean J, Hackett G, et al. Erectile dysfunction and coronary artery disease prediction: evidence-based guidance and consensus. Int J Clin Pract. 2010;64(7):848–57. 10.1111/j.1742-1241.2010.02410.x. 20584218 10.1111/j.1742-1241.2010.02410.x

[16] Diaconu CC, Manea M, Marcu DR, Socea B, Spinu AD, Bratu OG. The erectile dysfunction as a marker of cardiovascular disease: a review. Acta Cardiol. 2020;75(4):286–92. 10.1080/00015385.2019.1590498. 30955454 10.1080/00015385.2019.1590498

[17] Kubin M, Wagner G, Fugl-Meyer AR. Epidemiology of erectile dysfunction. Int J Impot Res. 2003;15(1):63–71. 10.1038/sj.ijir.3900949. 12605242 10.1038/sj.ijir.3900949

[18] Araujo AB, Durante R, Feldman HA, Goldstein I, McKinlay JB. The relationship between depressive symptoms and male erectile dysfunction: cross-sectional results from the Massachusetts male aging study. Psychosom Med. 1998;60(4):458–65. 10.1097/00006842-199807000-00011. 9710291 10.1097/00006842-199807000-00011

[19] Herder T, Spoelstra SK, Peters AWM, Knegtering H. Sexual dysfunction related to psychiatric disorders: a systematic review. J Sex Med. 2023;20(7):965–76. 10.1093/jsxmed/qdad074. 37279603 10.1093/jsxmed/qdad074

[20] Basson R, Rees P, Wang R, Montejo AL, Incrocci L. Sexual function in chronic illness. J Sex Med. 2010;7(1 Pt 2):374–88. 10.1111/j.1743-6109.2009.01621.x. 20092445 10.1111/j.1743-6109.2009.01621.x

[21] Politis M, Loane C, Wu K, et al. Neural response to visual sexual cues in dopamine treatment-linked hypersexuality in Parkinson’s disease. Brain. 2013;136(Pt 2):400–11. 10.1093/brain/aws326. 23378222 10.1093/brain/aws326

[22] Puth MT, Weckbecker K, Schmid M, Münster E. Prevalence of multimorbidity in Germany: impact of age and educational level in a cross-sectional study on 19,294 adults. BMC Public Health. 2017;17(1):826. 10.1186/s12889-017-4833-3. 29047341 10.1186/s12889-017-4833-3 PMC5648462

[23] Salonia A, Bettocchi C, Boeri L, Capogrosso P, Carvalho J, Cilesiz NC, et al. European association of urology guidelines on sexual and reproductive health-2021 update: male sexual dysfunction. Eur Urol. 2021;80(3):333–57. 10.1016/j.eururo.2021.06.007. 34183196 10.1016/j.eururo.2021.06.007

[24] Carter J, Lacchetti C, Andersen BL, Barton DL, Bolte S, Damast S, et al. Interventions to address sexual problems in people with cancer: American Society of Clinical Oncology clinical practice guideline adaptation of Cancer Care Ontario guideline. J Clin Oncol. 2018;36(5):492–511. 10.1200/JCO.2017.75.8995. 29227723 10.1200/JCO.2017.75.8995

[25] Gupta BP, Murad MH, Clifton MM, Prokop L, Nehra A, Kopecky SL. The effect of lifestyle modification and cardiovascular risk factor reduction on erectile dysfunction: a systematic review and meta-analysis. Arch Intern Med. 2011;171(20):1797–803. 10.1001/archinternmed.2011.440. 21911624 10.1001/archinternmed.2011.440

[26] Perelman MA. The sexual tipping point®: a mind/body model for sexual medicine. J Sex Med. 2009;6(3):629–32. 10.1111/j.1743-6109.2008.01177.x. 19210711 10.1111/j.1743-6109.2008.01177.x

[27] Berner M, Günzler C. Efficacy of psychosocial interventions in men and women with sexual dysfunctions-a systematic review of controlled clinical trials: part 1-the efficacy of psychosocial interventions for male sexual dysfunction. J Sex Med. 2012;9(12):3089–107. 10.1111/j.1743-6109.2012.02970.x. 23088533 10.1111/j.1743-6109.2012.02970.x

[28] Frühauf S, Gerger H, Schmidt HM, Munder T, Barth J. Efficacy of psychological interventions for sexual dysfunction: a systematic review and meta-analysis. Arch Sex Behav. 2013;42(6):915–33. 10.1007/s10508-012-0062-0. 23559141 10.1007/s10508-012-0062-0

[29] Zarski AC, Velten J, Knauer J, Berking M, Ebert DD. Internet- and mobile-based psychological interventions for sexual dysfunctions: a systematic review and meta-analysis. NPJ Digit Med. 2022;5(1):139. 10.1038/s41746-022-00670-1. 36085306 10.1038/s41746-022-00670-1 PMC9463146

[30] Velten J, Pantazidis P, Benecke A, Bräscher AK, Fehm L, Fladung AK, et al. Wie häufig werden Diagnosen aus dem Bereich der sexuellen Funktionsstörungen an deutschen Hochschulambulanzen für Psychotherapie an psychologischen Instituten vergeben? [Diagnosing sexual dysfunctions: how frequently are they assigned in German University outpatient clinics for psychotherapy?]. Z Sex Forsch. 2021;34(01):5–14. 10.1055/a-1362-2243.

[31] Mark KP, Arenella K, Girard A, Herbenick D, Fu J, Coleman E. Erectile dysfunction prevalence in the United States: report from the 2021 National Survey of Sexual Wellbeing. J Sex Med. 2024;21(4):296–303. 10.1093/jsxmed/qdae008. 38410029 10.1093/jsxmed/qdae008

[32] Moreira ED Jr, Hartmann U, Glasser DB, Gingell C; GSSAB Investigators Group. A population survey of sexual activity, sexual dysfunction and associated help-seeking behavior in middle-aged and older adults in Germany. Eur J Med Res. 2005;10(10):434–43. Available from: https://www.researchgate.net/publication/7480102_A_population_survey_of_sexual_activity_sexual_dysfunction_and_associated_help-seeking_behavior_in_middle-aged_and_older_adults_in_Germany 16287605

[33] Porst H, Montorsi F, Rosen RC, Gaynor L, Grupe S, Alexander J. The premature ejaculation prevalence and attitudes (PEPA) survey: prevalence, comorbidities, and professional help-seeking. Eur Urol. 2007;51(3):816–24. 10.1016/j.eururo.2006.07.004. 16934919 10.1016/j.eururo.2006.07.004

[34] Buddeberg C, Bucher T, Hornung R. Erektile Dysfunktion bei Männern in der zweiten Lebenshälfte. Urologe A. 2005;44(9):1045–51. 10.1007/s00120-005-0841-5. 15947903 10.1007/s00120-005-0841-5

[35] Grabski B, Kasparek K, Koziara K, Mijas M. Professional help-seeking in men experiencing sexual problems – the role of sexual identity and minority stress. J Sex Med. 2022;19(7):1090–7. 10.1016/j.jsxm.2022.04.012. 35654717 10.1016/j.jsxm.2022.04.012

[36] Nicolosi A, Buvat J, Glasser DB, Hartmann U, Laumann EO, Gingell C, et al. Sexual behaviour, sexual dysfunctions and related help seeking patterns in middle-aged and elderly Europeans: the global study of sexual attitudes and behaviors. World J Urol. 2006;24(4):423–8. 10.1007/s00345-006-0088-9. 16850339 10.1007/s00345-006-0088-9

[37] Baunacke M, Groeben C, Borkowetz A, Hoffmann F, Chun FKH, Weissbach L, et al. Urologist communication is a primary factor leading to erectile dysfunction treatment postprostatectomy. J Sex Med. 2024;21(10):904–11. 10.1093/jsxmed/qdae105. 39214554 10.1093/jsxmed/qdae105

[38] Perelman M, Shabsigh R, Seftel A, Althof S, Lockhart D. Attitudes of men with erectile dysfunction: a cross-national survey. J Sex Med. 2005;2(3):397–406. 10.1111/j.1743-6109.2005.20355.x. 16422872 10.1111/j.1743-6109.2005.20355.x

[39] Kliesch S, Cremers JF, Krallmann C, Epplen R, Scheffer B, Schubert T, et al. App-based therapy of erectile dysfunction using a digital health application (EDDIG study): a randomized, single-blind, controlled trial. Eur Urol Focus. 2024;10(6):1003–10. 10.1016/j.euf.2024.05.020. 38853028 10.1016/j.euf.2024.05.020

[40] El-Jawahri A, Reese JB, Traeger L, Dizon D, Cutler C, Bober S, et al. A digital intervention to address sexual health in hematopoietic stem cell transplant survivors. J Natl Compr Canc Netw. 2025;23(2):e247076. 10.6004/jnccn.2024.7076. 39938468 10.6004/jnccn.2024.7076

[41] Meyerowitz-Katz G, Ravi S, Arnolda L, Feng X, Maberly G, Astell-Burt T. Rates of attrition and dropout in app-based interventions for chronic disease: systematic review and meta-analysis. J Med Internet Res. 2020;22(9):e20283. 10.2196/20283. 32990635 10.2196/20283 PMC7556375

[42] Forschungsdatenzentrum der Statistischen Ämter des Bundes und der Länder (FDZ). Codebook Microcensus 2014 FDZ1. Version 5.2.2.0. Wiesbaden: FDZ; 2014. 10.21242/12211.2014.00.00.1.2.1.

[43] Kronthaler SM, Tissen-Diabaté T, Karsten MM, et al. Assessment of mental and chronic health conditions as determinants of health care needs and digital innovations for women with sexual dysfunction: cross-sectional population-based survey study in Germany. J Particip Med. 2025;17:e71301. 10.2196/71301. 40865091 10.2196/71301 PMC12386550

[44] Kronthaler SM, Tissen-Diabaté T, Neymeyer J, Blohmer J, Beier KM, Hatzler L. Prevalence of sexual dysfunctions and healthcare needs of transgender and gender-diverse people: results from a representative cross-sectional survey in Germany [abstract]. J Sex Med. 2024;21(Suppl 5):qdae054.011. 10.1093/jsxmed/qdae054.011.

[45] Derogatis LR, Revicki DA, Rosen RC, Jordan R, Lucas J, Spana C. Psychometric validation of the female sexual distress scale-desire/arousal/orgasm. J Patient Rep Outcomes. 2021;5(1):100. 10.1186/s41687-021-00359-1. 34559353 10.1186/s41687-021-00359-1 PMC8463644

[46] Velten J, Zarski AC. Therapie-Tools: Sexuelle Funktionsstörungen. Weinheim: Beltz; 2022. Available from: https://www.beltz.de/fachmedien/psychologie/produkte/details/48658-therapie-tools-sexuelle-funktionsstoerungen.html

[47] Wongpakaran N, DeMaranville J, Wongpakaran T. Validation of the relationships questionnaire (RQ) against the experience of close relationship-revised questionnaire in a clinical psychiatric sample. Healthcare. 2021;9(9):1174. 10.3390/healthcare9091174. 34574948 10.3390/healthcare9091174 PMC8470855

[48] Hill A. Sexualität in Zeiten des Internet. Psychotherapeut. 2011;56(6):475–84.

[49] Walther A, Mahler F, Debelak R, Ehlert U. Psychobiological protective factors modifying the association between age and sexual health in men: findings from the men’s health 40+ study. Am J Mens Health. 2017;11(3):737–47. 10.1177/1557988316689238. 28413941 10.1177/1557988316689238 PMC5675228

[50] van den Bussche H, Schön G, Kolonko T, Hansen H, Wegschneider K, Glaeske G, et al. Patterns of ambulatory medical care utilization in elderly patients with special reference to chronic diseases and multimorbidity–results from a claims data based observational study in Germany. BMC Geriatr. 2011;11:54. 10.1186/1471-2318-11-54. 21914191 10.1186/1471-2318-11-54 PMC3180370

[51] Quinaz Romana G, Kislaya I, Cunha Gonçalves S, Salvador MR, Nunes B, Matias DC. Healthcare use in patients with multimorbidity. Eur J Public Health. 2020;30(1):16–22. 10.1093/eurpub/ckz118. 31978229 10.1093/eurpub/ckz118

[52] Iroegbu C, Tuot DS, Lewis L, Matura LA. The influence of patient-provider communication on self-management among patients with chronic illness: a systematic mixed studies review. J Adv Nurs. 2025;81(4):1678–99. 10.1111/jan.16492. 39340765 10.1111/jan.16492 PMC11896829

[53] Berkman ND, Sheridan Sl, Donahue KE, Donahue Ke, Halpern DJ, Halpern Dj, et al. Low health literacy and health outcomes: an updated systematic review. Ann Intern Med. 2011;155(2):97–107. 10.7326/0003-4819-155-2-201107190-00005. 21768583 10.7326/0003-4819-155-2-201107190-00005

[54] Traumer L, Jacobsen MH, Laursen BS. Patients’ experiences of sexuality as a taboo subject in the Danish healthcare system: a qualitative interview study. Scand J Caring Sci. 2019;33(1):57–66. 10.1111/scs.12600. 30320477 10.1111/scs.12600

[55] Schomerus G, Matschinger H, Angermeyer MC. The stigma of psychiatric treatment and help-seeking intentions for depression. Eur Arch Psychiatry Clin Neurosci. 2009;259(5):298–306. 10.1007/s00406-009-0870-y. 19224105 10.1007/s00406-009-0870-y

[56] Al-Shaiji TF. Breaking the ice of erectile dysfunction taboo: a focus on clinician-patient communication. J Patient Exp. 2022;9 10.1177/23743735221077512 10.1177/23743735221077512 PMC8808006 35128040

[57] Mojtabai R, Evans-Lacko S, Schomerus G, Thornicroft G. Attitudes toward mental health help seeking as predictors of future help-seeking behavior and use of mental health treatments. Psychiatr Serv. 2016;67(6):650–7. 10.1176/appi.ps.201500164. 26876662 10.1176/appi.ps.201500164

[58] Pretorius C, Mccashin D, Kavanagh NC, Coyle D. Searching for mental health: a mixed-methods study of young people's online help-seeking. Proc CHI Conf Hum Factors Comput Syst. 2020;1-13. 10.1145/3313831.3376328

[59] Doll CM, Michel C, Rosen M, Osman N, Schimmelmann BG, Schultze-Lutter F. Predictors of help-seeking behaviour in people with mental health problems: a 3-year prospective community study. BMC Psychiatry. 2021;21(1):432. 10.1186/s12888-021-03435-4. 34479537 10.1186/s12888-021-03435-4 PMC8414662

[60] Madeira CR, Tonin FS, Fachi MM, Borba HH, Ferreira VL, Leonart LP, et al. Efficacy and safety of oral phosphodiesterase 5 inhibitors for erectile dysfunction: a network meta-analysis and multicriteria decision analysis. World J Urol. 2021;39(3):953–62. 10.1007/s00345-020-03233-9. 32388784 10.1007/s00345-020-03233-9

[61] Organisation for Economic Co-operation and Development (OECD), Health at a Glance 2023: OECD Indicators, Paris: OECD Publishing; 2023. 10.1787/7a7afb35-en.

[62] Kirana PS, Gudeloglu A, Sansone A, Fode M, Reisman Y, Corona G, et al. E-sexual health: a position statement of the European Society for Sexual Medicine. J Sex Med. 2020;17(7):1246–53. 10.1016/j.jsxm.2020.03.009. 32340920 10.1016/j.jsxm.2020.03.009

[63] Sewak A, Yousef M, Deshpande S, Seydel T, Hashemi N. The effectiveness of digital sexual health interventions for young adults: a systematic literature review (2010–2020). Health Promot Int. 2023;38(1):daac104. 10.1093/heapro/daac104. 36757346 10.1093/heapro/daac104

[64] Andersson E, Walén C, Hallberg J, Paxling B, Dahlin M, Almlöv J, et al. A randomized controlled trial of guided internet-delivered cognitive behavioral therapy for erectile dysfunction. J Sex Med. 2011;8(10):2800–9. 10.1111/j.1743-6109.2011.02391.x. 21797983 10.1111/j.1743-6109.2011.02391.x

[65] McCabe MP, Price E, Piterman L, Lording D. Evaluation of an internet-based psychological intervention for the treatment of erectile dysfunction. Int J Impot Res. 2008;20(3):324–30. 10.1038/ijir.2008.3. 18305485 10.1038/ijir.2008.3

[66] Schover LR, Canada AL, Yuan Y, Sui D, Neese L, Jenkins R, et al. A randomized trial of internet-based versus traditional sexual counseling for couples after localized prostate cancer treatment. Cancer. 2012;118(2):500–9. 10.1002/cncr.26308. 21953578 10.1002/cncr.26308 PMC4022136

[67] Altin SV, Stock S. Impact of health literacy, accessibility and coordination of care on patient’s satisfaction with primary care in Germany. BMC Fam Pract. 2015;16:148. 10.1186/s12875-015-0372-0. 26492959 10.1186/s12875-015-0372-0 PMC4619202

[68] Goldstein I, Komisaruk BR, Pukall CF, et al. International Society for the Study of Women’s Sexual Health (ISSWSH) review of epidemiology and pathophysiology, and a consensus nomenclature and process of care for the management of persistent genital arousal disorder/genito-pelvic dysesthesia (PGAD/GPD). J Sex Med. 2021;18(4):665–97. 10.1016/j.jsxm.2021.01.172. 33612417 10.1016/j.jsxm.2021.01.172

[69] Eisenberg D, Downs MF, Golberstein E, Zivin K, Zivin K. Stigma and help seeking for mental health among college students. Med Care Res Rev. 2009;66(5):522–41. 10.1177/1077558709335173. 19454625 10.1177/1077558709335173

[70] Beier KM, Loewit K. Sexual medicine in clinical practice. New York: Springer; 2012. 10.1007/978-1-4641-4421-3.

[71] Brotto LA, Atallah S, Carvalho J, Gordon E, Pascoal PM, Reda M, et al. Psychological and interpersonal dimensions of sexual function and dysfunction: recommendations from the fifth international consultation on sexual medicine (ICSM 2024). Sex Med Rev. 2025;13(2):118–43. 10.1093/sxmrev/qeae073. 39786497 10.1093/sxmrev/qeae073

[72] Bouchard KN, Bergeron S, Rosen NO. Feasibility of a cognitive-behavioral couple therapy intervention for sexual interest/arousal disorder. J Sex Res. 2025;62(5):765–75. 10.1080/00224499.2024.2333477. 38593203 10.1080/00224499.2024.2333477

[73] Mielck A, Vogelmann M, Leidl R, Leidl R. Health-related quality of life and socioeconomic status: inequalities among adults with a chronic disease. Health Qual Life Outcomes. 2014;12:58. 10.1186/1477-7525-12-58. 24761773 10.1186/1477-7525-12-58 PMC4011770

